# Multimodal LLM-driven IoT digital healthcare platform for intelligent dysphagia dietary monitoring

**DOI:** 10.3389/fnut.2026.1829703

**Published:** 2026-06-29

**Authors:** Zebang He, Steve W. Y. Mung, Anna Chi Shan Kam, Chetwyn C. H. Chan

**Affiliations:** 1 Research and Development Office, The Education University of Hong Kong, New Territories, Hong Kong SAR, China; 2 Department of Special Education and Counselling, The Education University of Hong Kong, New Territories, Hong Kong SAR, China; 3 Department of Psychology, The Education University of Hong Kong, New Territories, Hong Kong SAR, China

**Keywords:** digital healthcare, dysphagia, international dysphagia diet standardisation initiative, internet of things, IoT-based dietary monitoring system, large language model, nutrition monitoring

## Abstract

**Introduction:**

Patients with dysphagia who are transitioning from clinical to domestic environments face significant life-threatening risks due to the “nutrition–texture disconnect”—the systemic gap between hospital-prescribed International Dysphagia Diet Standardisation Initiative (IDDSI) levels and actual home-prepared meals. Existing dietary monitoring tools effectively track caloric intake, yet they lack the rheological sensing capabilities required to prevent acute choking and liquid aspiration hazards. This preliminary feasibility study introduces a novel, smartphone-centric Internet of Things platform that operationalizes a multimodal large language model (MLLM) as an “intelligent soft sensor” to bridge this safety gap.

**Methods:**

The system utilizes a three-stage cascaded pipeline—incorporating chain-of-thought reasoning and bi-directional safety logic—to perform automated dish recognition, localized nutritional retrieval, and pre-consumption IDDSI texture auditing from a single smartphone image. To evaluate performance, the platform was benchmarked against the newly established Clinical-Expert IDDSI Validation Benchmark, a curated dataset of 115 diverse meal images independently verified via physical rheological testing by certified clinical specialists. A comprehensive comparative evaluation was conducted across 14 commercial and open-source models.

**Results:**

Our Qwen-based pipeline achieved a total exact match accuracy of 61.74%. Crucially, the system demonstrated the highest safety profile among 14 tested commercial and open-source models, achieving the lowest total false negative rate (FNR) of 9.2%. While localized open-source models exhibited a critical failure mode regarding liquid aspiration hazards (100% FNR), our cloud-based system maintained a robust 7.14% FNR for liquids.

**Discussion:**

These empirical results indicate that the proposed platform serves as a superior, safety-first screening proxy tool for digital healthcare. By prioritizing a low hazard–miss rate over raw conversational baseline accuracy, the system successfully bridges the safety gap between clinical prescriptions and daily home-based monitoring. The framework offers an ethical, non-invasive, and highly scalable soft-sensing solution for longitudinal, unsupervised dysphagia oversight in home-care settings.

## 1 Introduction

Dysphagia, defined as an impairment in moving saliva, liquid, or food safely from the mouth to the stomach (1), significantly compromises the health and quality of life across an individual’s lifespan. Recent clinical reviews (2) highlight the critical association between dysphagia and severe adverse outcomes, including malnutrition, dehydration, aspiration pneumonia, prolonged hospitalization, and elevated mortality risks. Given the burden associated with the care and cost of dysphagia, early identification and effective dietary monitoring are urgent public health priorities, specifically within the context of aging, post-stroke recovery, and neurodegenerative diseases.

To standardize dietary monitoring, the International Dysphagia Diet Standardisation Initiative (IDDSI) provides a globally adopted framework for texture-modified foods and thickened liquids. The framework categorizes items into eight standardized levels (0–7) verified through simple methods such as flow and fork pressure tests (3). By establishing a common lexicon, IDDSI reduces ambiguity in prescribing safe consistencies and facilitates consistent quality control across care settings. Furthermore, implementation studies have shown that IDDSI levels correspond to measurable rheological and mechanical properties essential for safe, individualized meal planning.

However, a significant practical challenge remains: The accurate application of IDDSI tests in real-world settings often relies heavily on clinical expertise. Nonspecialists, including family caregivers and patients in domestic settings, frequently misinterpret visual cues or perform manual checks inconsistently, leading to dangerous variability in food classification. Recent meta-analyzes have questioned the reproducibility of these manual tests outside of specialist teams (4). While purpose-built tools such as the IDDSI framework aim to reduce variation, misclassification remains a strong risk without an experienced clinician present at every meal.

Despite the urgency of this problem, current technological interventions have been unable to bridge this gap. Mainstream diet tracking applications utilize artificial intelligence (AI) for calorie and macronutrient estimation, but they systematically overlook the texture and flow properties that determine swallowing safety (5). This “nutrition–texture disconnect” leaves patients vulnerable; a user may meet their nutritional targets digitally while unknowingly consuming textures that pose an acute aspiration or choking risk. Furthermore, most existing systems treat a plate as a single entity, lacking the granular localization required to identify specific unsafe components within a mixed meal.

Addressing these limitations requires a diagnostic aid that can provide a “second opinion” at the point of consumption. With a standard smartphone camera functioning as an intelligent soft sensor, the advanced reasoning capabilities of multimodal large language models (MLLMs) are utilized to simulate a clinician’s visual audit. Such a sensor will operate within a robust Internet of Things (IoT) ecosystem, ensuring that data captured at the edge (the patient) are processed with cloud intelligence and returned in near-real time to deliver safety-critical recommendations. This approach seeks to reconcile nutritional adequacy with mechanical safety, providing a non-invasive, automated safety loop that extends medical-grade oversight from the hospital ward to the domestic dining table.

In this preliminary feasibility study, we propose an MLLM-driven dietary IoT platform that works as a non-invasive intelligent soft sensor. The system operates the IDDSI framework at the point of consumption without the need for auxiliary hardware or specialized rheological tools. The operational workflow initiates when a user captures a meal image via the mobile interface, which is securely transmitted to a cloud-based layer for processing through a cascaded MLLM pipeline. The first stage serves as a semantic transduction sensor, performing instance-level dish identification and segmentation. Once localized, the system queries the Centre for Food Safety (CFS) of Hong Kong to retrieve culturally relevant nutritional data. The third stage is engineered as an IDDSI expert reasoning module, wherein bi-directional safety logic is utilized to derive texture classifications and formulate actionable modification advice.

Our evaluation against a newly established expert benchmark demonstrates that while raw exact-match accuracy remains a challenge for frontier MLLMs, our safety-optimized pipeline—driven by the Qwen API—achieves a significantly lower total false negative rate (FNR; 9.2%) compared with other frontier models. Notably, while localized open-source models failed entirely on liquid aspiration risks, our system maintained a robust 7.14% FNR for liquids. The major contributions of this study are as follows:

1. Development of an MLLM-driven “intelligent soft sensor”: A method for generating integrated nutritional data and IDDSI classifications from a single user interaction is introduced. This is achieved through a cloud-edge IoT architecture that bridges the gap between optical data and mechanical food properties.
2. Implementation of bi-directional safety logic: A cascaded MLLM pipeline is proposed and created to address the inverse risk profiles of dysphagia, through which the underestimation of solid food hardness (choking risk) and the overestimation of liquid viscosity (aspiration risk) are minimized.
3. Establishment of the clinical-expert IDDSI validation (CEIV) benchmark: The performance of leading MLLMs is evaluated quantitatively using a curated dataset of 115 images verified by clinical specialists. The results demonstrate the feasibility of AI-based safety screening as a preliminary tool for home-based dysphagia care.

This paper is organized as follows: Section II reviews the related research on dysphagia and AI-driven dietary monitoring. Section III details the proposed system architecture, the cascaded MLLM methodology, and prompt engineering techniques. Section IV presents the prototype implementation and quantitative validation results against the expert benchmark. Section V discusses clinical implications, the “modality–construct gap,” and limitations. Finally, Section VI concludes the study and outlines future research directions.

## 2 Related work

### 2.1 Literature search strategy

A systematic search was conducted across three primary academic repositories—PubMed/MEDLINE, IEEE Xplore, and Google Scholar—to ensure the scientific rigor of this review. The search period was restricted to the last decade (2016–2026) to capture the transition from classical convolutional neural networks (CNNs) to modern MLLMs and the global adoption of the IDDSI framework.

The search utilized boolean strings combining terms from three domains:

- Clinical: “Dysphagia,” “Swallowing Disorders,” “Aspiration Pneumonia,” and “IDDSI”
- Technical: “Vision-Language Models (VLM),” “Multimodal Large Language Models (MLLM),” “Intelligent Soft Sensors,” and “Edge-Cloud IoT”
- Application: “Food Texture Analysis,” “Automated Dietary Monitoring,” and “Clinical Decision Support”

A total of 142 records were initially screened, with 38 studies selected for in-depth analysis and inclusion on the basis of their specific relevance to automated texture assessment or clinical decision support in dysphagia management.

### 2.2 AI-driven clinical decision support in dysphagia

Dysphagia research has evolved significantly from early physiological studies to the standardized era of IDDSI, enabling robust cross-study comparisons (6–10). AI transforms dysphagia management across two primary domains: imaging and sensing.

In the imaging domain, deep learning models automate the interpretation of videofluoroscopic swallowing studies (VFSS) and fiberoptic endoscopic evaluation of swallowing (FEES). These advancements enable faster and more consistent detection of aspiration and penetration, reducing the diagnostic burden on clinicians (11–14). In the sensing domain, previous studies applied machine learning to non-invasive modalities—including accelerometry, surface electromyography, and cervical auscultation—to facilitate remote risk assessment (14). While recent trials have confirmed the feasibility of wearable devices for longitudinal monitoring (15), and emerging audio-based models trained on cough or speech signals can effectively predict aspiration risk (16), these modalities provide active physiological monitoring during or after the act of swallowing only. They do not address the pre-consumption audit of food texture—a critical gap in the safety loop.

Large language models (LLMs) are a rapid development frontier in this domain, showing potential in speech language pathology for drafting therapy plans and clinical narratives (17). They can extract information from unstructured clinical notes, thus being a powerful extraction layer for diverse patient data. However, current LLM research in dysphagia focuses nearly exclusively on text-based screening and risk prediction. No existing research applies MLLMs to the visual analysis and mechanical auditing of actual food consumed by patients.

### 2.3 Evolution of food analysis: from CNNs to MLLMs

General dietary assessment has shifted from labor-intensive manual logging to automated, image-driven pipelines supported by comprehensive nutritional databases (18, 19). Conventional computer vision architectures primarily leverage CNNs for dish localization, menu-aware recognition, and volumetric estimation (20–25). To enhance clinical utility and convenience, modern systems increasingly integrate artificial IoT platforms (AIoT) or edge computing frameworks to facilitate real-life monitoring in cluttered backgrounds (5, 26–29).

Despite these technical strides, CNN-based systems are inherently “closed-vocabulary,” often struggling with the high textural variance of regional cuisines and novel food presentations. The emergence of vision-language models and MLLM represents a new development direction. Unlike traditional classifiers, MLLMs process visual and textual tokens in a shared semantic space, allowing them to infer hidden attributes—such as whether a dish is fibrous, pureed, or thin—by leveraging massive pre-trained world knowledge. This capability allows the MLLM to function as an intelligent soft sensor, estimating hard-to-measure rheological properties from standard RGB image data.

### 2.4 Nutrition–texture disconnect in digital health

Mainstream diet tracking applications have a fundamental clinical gap: They prioritize calories and macronutrients while disregarding the mechanical flow properties essential for swallowing safety (5). This “nutrition–texture disconnect” makes existing digital health tools insufficient—and potentially unsafe—for dysphagia management.

Most contemporary systems consider a plate a single entity, lacking the granular, instance-level localization required to identify specific unsafe components within a mixed meal. By terminating at a simple nutrient lookup, these pipelines fail to offer the actionable, IDDSI-oriented texture modification advice required for a complete safety loop. Our platform addresses this disconnect by reconciling nutritional adequacy with mechanical safety. It utilizes a modular architecture that supports the “hot-swapping” of regional nutritional databases while maintaining a universal, MLLM-driven clinical safety audit logic.

## 3 Proposed dietary platform

The proposed platform is engineered as a cloud-based IoT ecosystem that transforms a standard smartphone into an intelligent soft sensor. Unlike traditional sensors that measure physical quantities through hardware transducers, this soft sensor utilizes a cascaded inference pipeline to derive complex clinical data from raw optical inputs.

To situate our findings within the current state of the art, Table 1 compares the proposed platform and previous digital health works.

**Table 1:** Comparison of the proposed platform and previous work.

References	Focus	Strengths	Limitations
Proposed platform	Single-photo workflow Dish-wise recognition and naming with LLM IDDSI-level classification and suggestion with LLM	Per-dish analysis of mixed plates Minimum input effort Includes IDDSI-related information	Dependent on LLM visual reliability Image-only IDDSI inference Not providing IDDSI suggestion for drinks Nutrition values depend on an external database
(19)	Single-image meal recognition by a CNN Image segmentation by DeepLab Volume and calorie estimation by using CNN	Early work on using deep learning network to map image to calories from images taken in a non-restaurant setting	High computational complexity Error accumulation across multiple deep learning processes Weak at fine-grained food recognition Only estimates calories
(18)	Single-photo workflow Food-wise recognition and segmentation via hierarchical segmentation Nutrition estimation	Mobile food recognition system Automatic food detection and recognition in real-life settings with cluttered backgrounds	Limited available number of food categories in recognition
(21)	Food recognition and segmentation in mixed dishes by using contextual relation networks	Provide solution for mixed dishes by using a contextual relation network	Focuses on food recognition only without nutrition analysis
(24)	Introduce Nutrition5k real-world food dishes with annotations	First large-scale dataset of dishes with corresponding video, depth images, component weights, and nutritional annotations	Performance relies heavily on depth information and prediction Dataset biased toward Western-style dishes
(35)	Portable and inexpensive rheometer for fluid viscosity quantification	Obtain viscosity through easy-to-use and portable devices	The IDDSI framework has not been implemented Considers liquids only
(37)	Deep learning- and IoT-based diet and nutrition monitoring system	One step for food recognition and nutrition monitoring Accurate and user-friendly food portion estimation	Lacks IDDSI-level classification and suggestion

### 3.1 Platform overview

#### 3.1.1 Technical orchestration

The core reasoning engine of this platform is conceptualized as an intelligent soft sensor (or virtual sensor). This framework aligns with the established engineering paradigm where a computational model is used to estimate hard-to-measure variables—specifically IDDSI texture levels and nutritional density—by processing easy-to-measure data, such as RGB pixels captured via standard mobile hardware. The platform utilizes the Qwen-VL-Max MLLM, implemented via a high-throughput API format. The framework is anchored to the 2026.4.30 model version to ensure the reproducibility of results and stability of the inference logic. This cloud-based approach allows the platform to offload intensive multimodal processing tasks—such as spatial reasoning and semantic mapping—to high-performance servers, enabling high-fidelity safety audits on consumer-grade mobile devices.

#### 3.1.2 Functional pipeline

**Figure 1:**
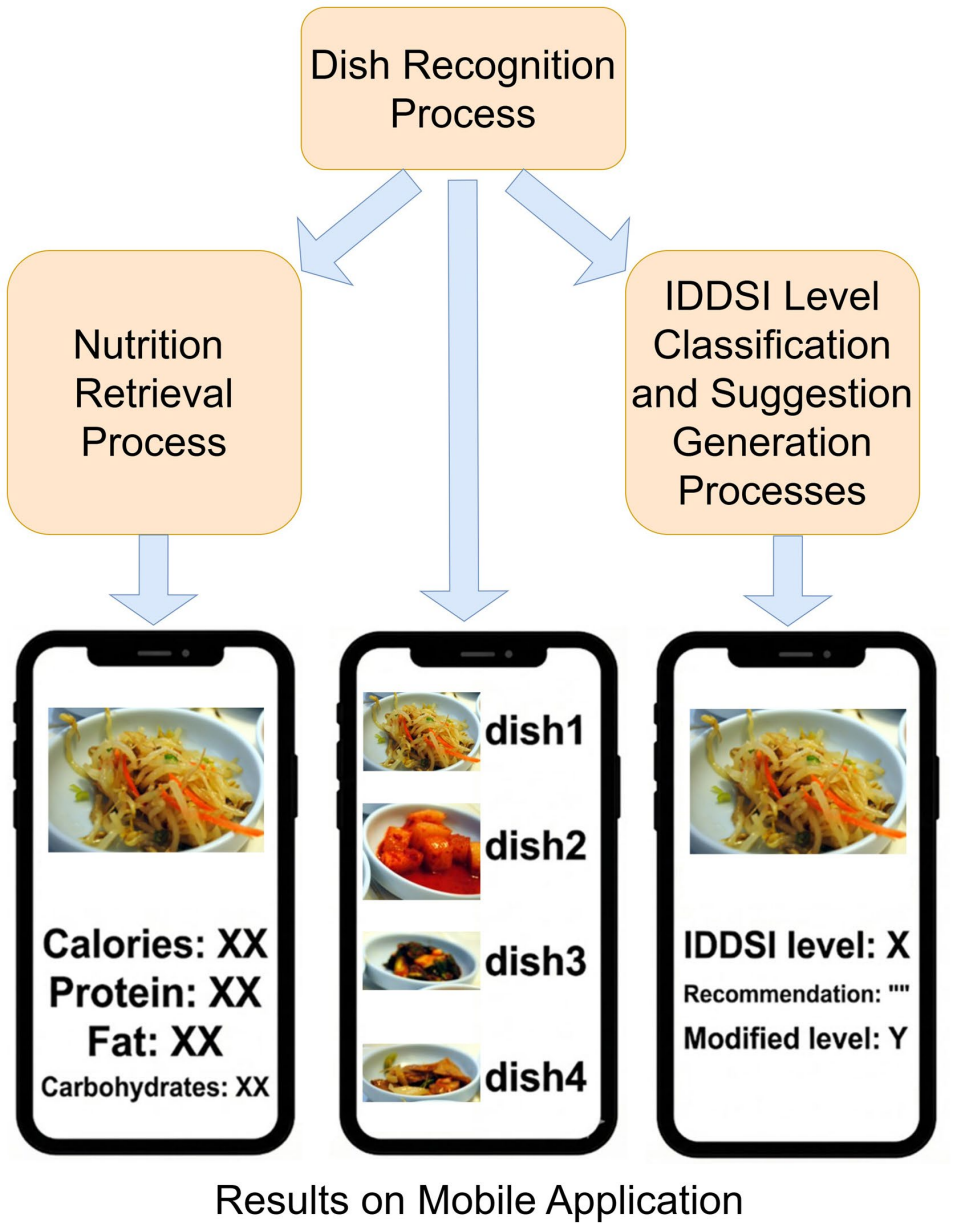
Block diagram of the proposed MLLM-driven dietary IoT platform.

The system architecture, illustrated in Figure 1, processes a single meal image to fulfill three primary functions:

1. Instance-level dish localization and semantic naming, which breaks down the meal into its constituent dietary components.
2. Automated nutritional retrieval, mapping the identified items to regional repositories (e.g., the CFS database) to generate a granular nutritional profile.
3. Safety-critical IDDSI texture audit, utilizing specialized prompt scaffolding to provide accurate texture predictions and modification suggestions at the point of consumption.

### 3.2 Stage 1: Dish recognition

**Figure 2:**
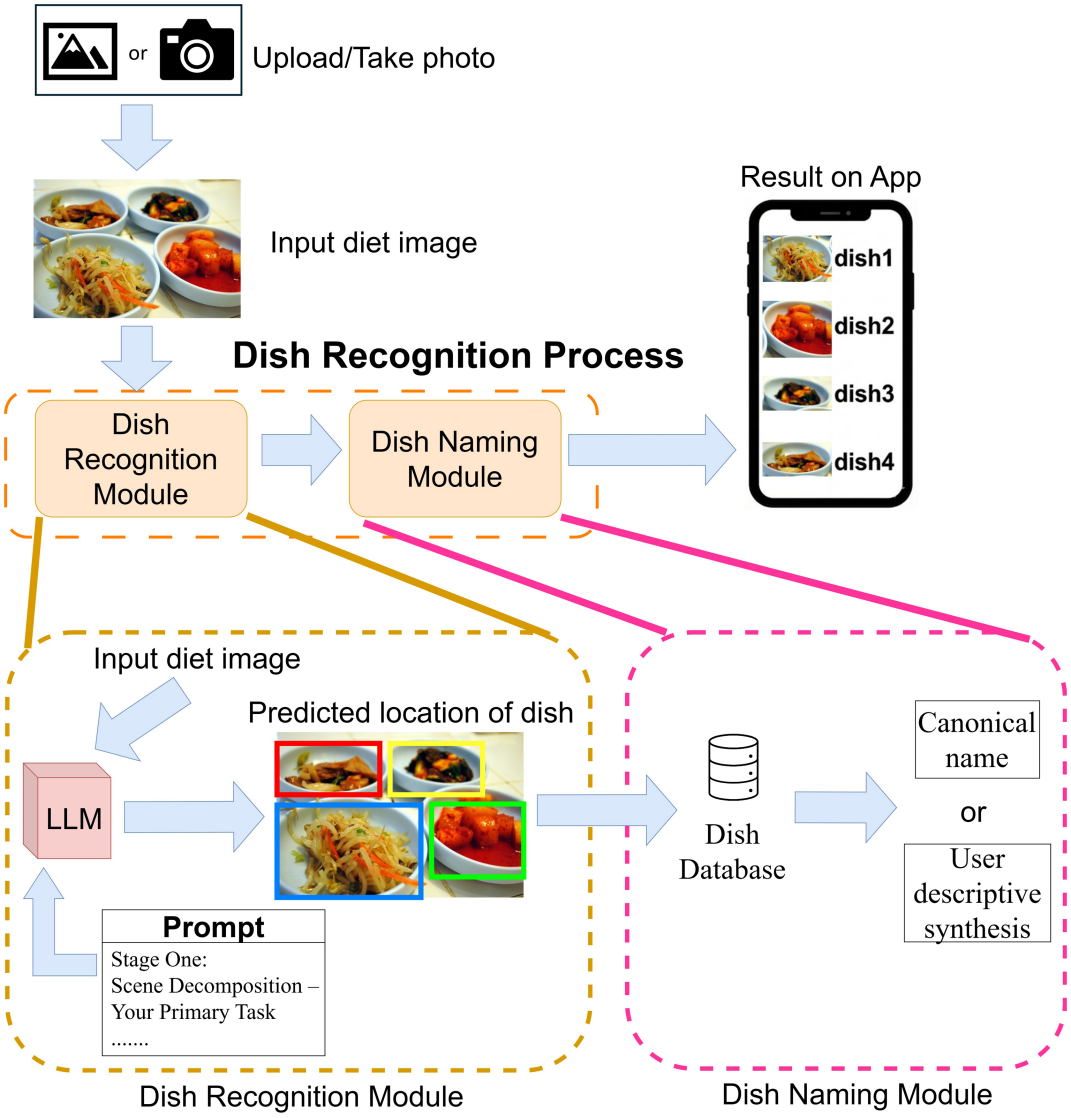
Workflow of dish recognition process.

The initial stage of the pipeline is dish recognition, which performs the spatial localization and semantic classification of culinary items (Figure 2). This stage functions as the transduction element of the soft sensor, converting raw RGB pixel data into a structured list of identified food objects.

#### 3.2.1 Hierarchical segmentation logic

To handle the complexity of multi-item plates, the MLLM employs a hierarchical decision tree to classify detected regions. For set meals, heterogeneous regions (e.g., a tray with multiple bowls) are segmented into distinct items to prevent data coarsening, where a single IDDSI level might otherwise be incorrectly applied to an entire meal despite varying textures. Conversely, for integrated dishes, cohesive items (e.g., stews or mixed salads) are treated as single entities to prevent the double-counting of nutritional values and ensure the IDDSI audit evaluates the composite texture.

#### 3.2.2 Naming and canonical prioritization

The naming module utilizes canonical prioritization strategy. By identifying a dish by its canonical name (e.g., “Hainanese chicken”), the model accesses a deeper semantic priority regarding its preparation. Certain names automatically trigger a high-risk choking food flag; for example, any item identified as containing skins, bones, or fibrous stalks is immediately prioritized for a Level 7 (Regular) classification to maintain a conservative safety bias. Furthermore, the model uses the identified dish name to simulate IDDSI physical constraints, such as evaluating “minced meat” against the Level 5 threshold (<4 mm lumps).

#### 3.2.3 Multitiered database fallback

To bridge the semantic gap between generated names and the fixed CFS Database taxonomy, the system employs three-tier fallback logic. First, the system utilizes a vector-based semantic search to retrieve the nearest nutritional neighbor if an exact string match fails. If confidence remains low, then the model performs ingredient-based decomposition, calculating a sum-of-parts nutritional estimate from identified constituents. Finally, in highly ambiguous cases, the interface triggers a user-mediated verification prompt to ensure data continuity and safety.

### 3.3 Stage 2: Nutrition retrieval

**Figure 3:**
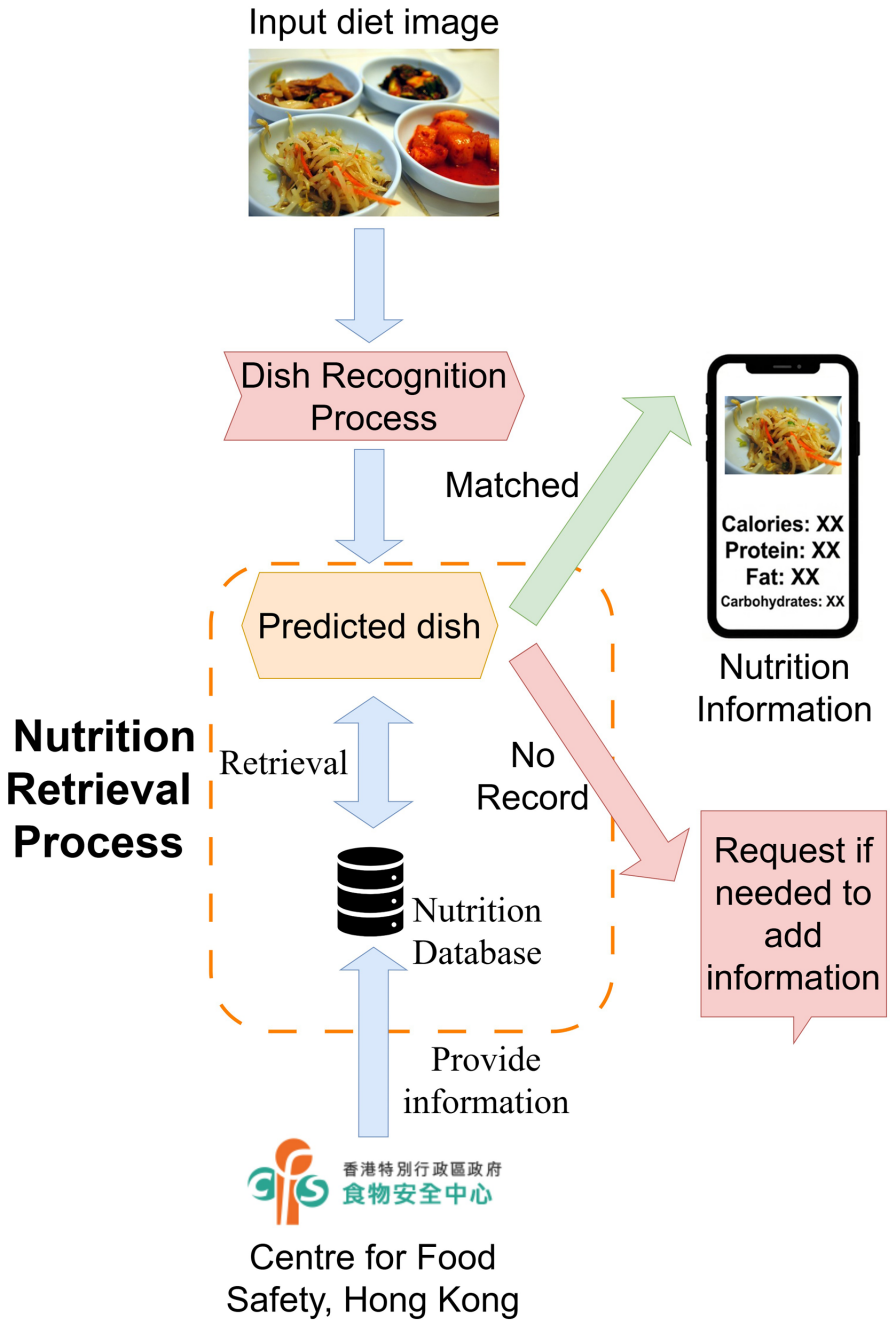
Workflow of the nutrition retrieval process.

Once the dish items are successfully localized and named, the system initiates a query to the external nutrition database managed by the CFS (Figure 3). Representative samples of the nutritional metadata retrieved from the CFS repository are provided in Table 2. This stage ensures that the retrieved data are chemically accurate and reflective of the specific culinary composition found within the local region.

**Table 2:** Selected samples of nutrition information from CFS.

Food name (English)	Food name (Chinese)	Energy (kcal)	Protein (g)	Carbohydrate (g)	Fat (g)	Dietary fiber (g)	Calcium (mg)	Iron (mg)
Indian crispy bread	印度薄脆餅	371	25.56	59.87	3.25	18.6	143	7.80
Green beans	四季豆	343	18.81	64.11	2.02	25.2	186	3.40
Cooked dried pink salmon	粉紅三文 (熟,乾煮)	149	25.56	0	4.42	0	17	0.99
Fresh pink salmon	粉紅三文魚(生)	116	19.94	0	3.45	0	13	0.77
Lean pork	豬肉(瘦)	143	20.30	1.5	6.20	0	6	3.00
Cantaloupe	哈蜜瓜	34	0.84	8.16	0.19	0.9	9	0.21

Nutritional values are normalized per 100 g of food.

#### 3.3.1 Nutritional mapping logic

The canonical dish name predicted by the MLLM is parsed and matched against CFS database entries to resolve naming variations. Upon a successful match, the system retrieves standardized nutritional values—including energy, protein, carbohydrates, and total fat—normalized per 100 g of food. It must be explicitly acknowledged that the current platform design solely provides these per-100 g reference values and does not attempt to estimate or calculate actual portion mass. Consequently, absolute nutritional intake figures reported by the platform carry an unquantified error that is directly proportional to the deviation of the user’s actual consumed portion size from the baseline 100 g database standard. This intentional design constraint sidesteps the volatile error distributions documented in contemporary single-view visual food portion estimation literature (30, 31), prioritizing deterministic, regional database integrity over high-overhead, unvalidated visual volume estimation. By leveraging the official CFS repository, the platform provides users with high-fidelity nutritional feedback that is culturally relevant to the Hong Kong population, establishing a baseline for dietary monitoring before the texture audit.

### 3.4 Stage 3: IDDSI level classification and suggestion generation

**Figure 4:**
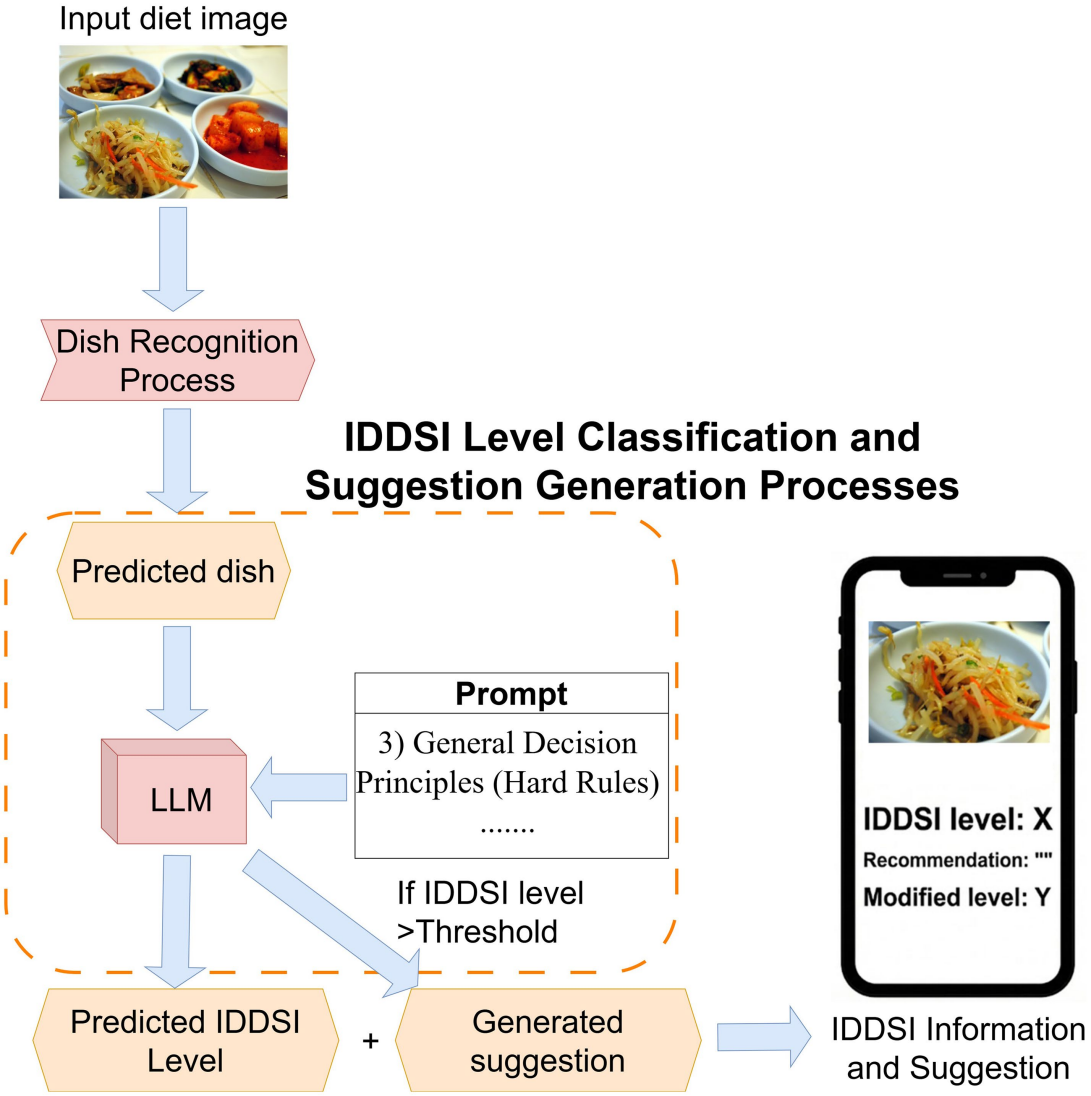
Workflow of IDDSI-level classification and suggestion generation processes.

Following the localization of items and the completion of the nutrition retrieval process (Figure 3), the system initiates the IDDSI level classification and suggestion generation (illustrated in Figure 4). The nutrition–texture disconnect is addressed by decoupling this stage from the purely generative naming functions and utilizes a modular inference layer focused on bi-directional risk management and safety-first suggestion generation.

#### 3.4.1 Bi-directional risk management

Unlike nutritional tracking, which is primarily additive, texture modification requires a specialized bi-directional logic to manage the inverse risk profiles of solids and liquids. For solid hazards (choking), the model prioritizes negative sensitivity, recognizing that higher IDDSI levels (Levels 4–7) represent higher risks and thus enforcing a conservative ceiling to prevent the underestimation of hardness. Conversely, for liquid hazards (aspiration), the model identifies lower IDDSI levels (low viscosity, Levels 0–2) as the primary risk factors. Our Qwen-based pipeline is specifically conditioned to detect optical markers associated with thin-liquid flow.

#### 3.4.2 Safety-first suggestion generation

Corrective suggestions are generated exclusively when the audited IDDSI level exceeds the user’s clinically prescribed safety threshold. The model identifies the minimal necessary modification (e.g., “Steam for 10 additional minutes” or “Apply Level 2 thickener”) required to ensure that the meal meets the target safety level. This process ensures that the intervention preserves both the nutritional density and the palatability of the meal while effectively closing the safety loop for the patient.

### 3.5 Prompt engineering scaffolding

**Figure 5:**
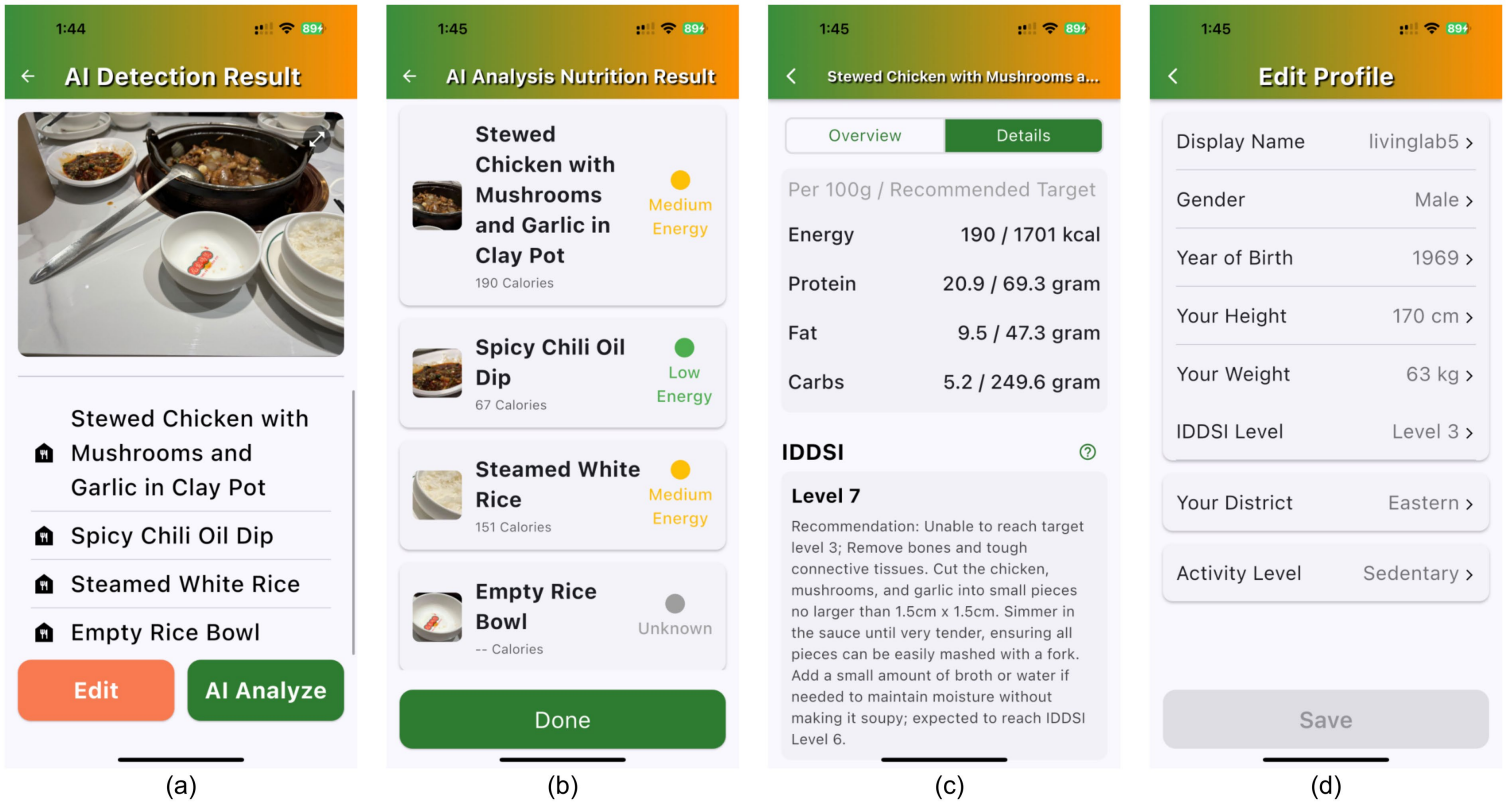
Prompt templates for the MLLM. **(a)** Prompt for multi-item dish recognition and segmentation and **(b)** prompt for IDDSI-level clinical evaluation and modification suggestion generation.

To support complex reasoning, enforce strict output schemas, and mitigate the stochastic behavior inherent in large-scale models, we employ three primary prompting strategies: chain-of-thought (CoT) (32), few-shot learning (33), and negative prompting (34). These techniques collectively ensure that the MLLM functions as a reliable intelligent soft sensor, translating raw visual data into structured clinical insights with high fidelity. A high-level mapping of these prompt engineering strategies to specific clinical objectives is summarized in Table 3, and the specific underlying logic and natural language structures used for these prompts are documented in Figure 5.

**Table 3:** Summary of prompt engineering scaffolding by pipeline stage.

Pipeline stage	Prompting technique	System application	Specific clinical objective
Stage 1: Semantic transduction	CoT (33)	Forces hierarchical breakdown (evaluating physical boundaries before naming)	Prevents data coarsening; ensures small, high-risk items on a mixed plate are not overlooked
	Few-shot learning (34)	Provides examples of canonical vs. descriptive naming logic	Ensures high-accuracy mapping to local dietary taxonomy (CFS database)
Stage 3: IDDSI safety audit	CoT (33)	Step-by-step simulation of IDDSI physical tests (e.g., simulating a spoon tilt test)	Forces the model to evaluate mechanical physics before assigning a final IDDSI safety level
	Few-shot learning (34)	Examples of strict JSON output formats and target-bounded modifications	Ensure the app code does not crash; limits modification advice to the patient’s specific prescription
	Negative prompting (35)	Explicitly forbids the underestimation of hardness or ignoring liquid risks	Minimizes FNR, suppresses conversational hallucinations, and enforces a conservative safety bias

CoT (33) is utilized to elicit intermediate reasoning steps, an essential technique for the multistep inference required in dysphagia care. In the initial sensing stages, CoT requires the model to categorize the scene structure—distinguishing between a set meal and an integrated dish—and determine the appropriate naming route before generating a result. Crucially, during the IDDSI classification stage, the inference chain forces the model to internally simulate a physical IDDSI test (e.g., a visual fork pressure or flow rate simulation) before finalizing a level assignment. This decomposition of complex visual tasks into verifiable logical sequences significantly reduces hallucinations and enhances the reliability of the clinical audit.

Few-shot learning (34) is employed to provide the model with a clear compliance framework for the required JSON output schema and specialized edge-case handling. By providing visual–textual benchmarks of integrated versus discrete meal components, these exemplars improve format adherence and label stability across the pipeline. In the suggestion generation stage, Few-shot learning ensures that clinical recommendations are practical and verifiable. The samples cover various dish levels relative to user targets, instructing the model on how to calculate the minimal modification required to ensure safety without over-processing the meal.

Finally, negative prompting (35) is a final defensive measure to suppress off-policy behavior and irrelevant conversational content. In our pipeline, explicit exclusion instructions are implemented to strictly prohibit recurring error patterns, such as the underestimation of IDDSI levels or the generation of extraneous explanations that would break the automated data flow. This strategy ensures that the model maintains a conservative safety profile and strictly adheres to the structured data requirements of the IoT platform, facilitating the seamless translation of MLLM reasoning into actionable digital healthcare monitoring.

## 4 Prototype demonstration and method validation

### 4.1 Mobile application overview

**Figure 6:**
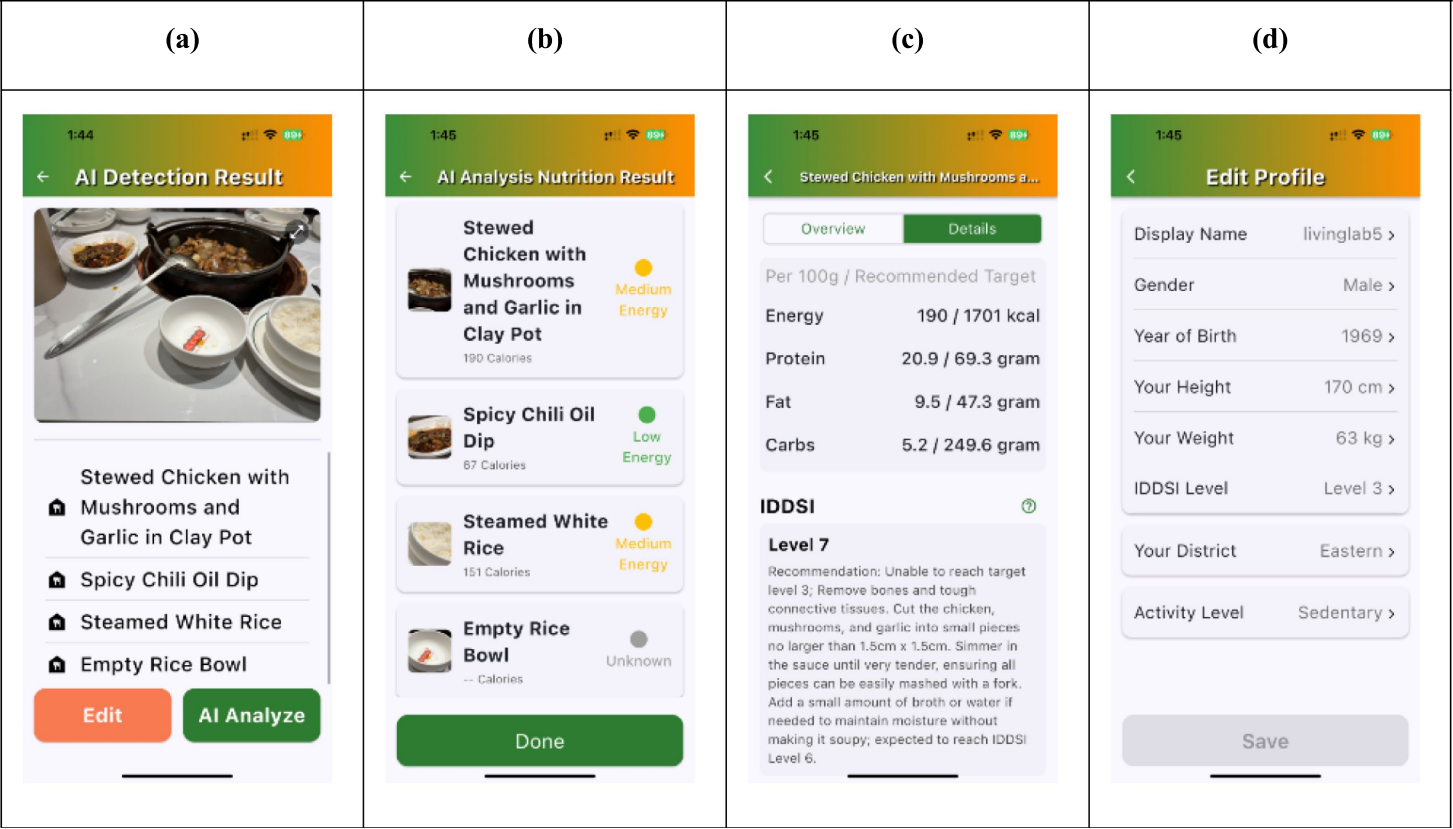
Overview of the mobile application user interface: **(a)** Main page; **(b)** analysis results for a captured meal, including dish identification; **(c)** nutrition information, and IDDSI-level classification and suggestion; and **(d)** personal information interface, allowing users to manage their profile and specifically configure their target IDDSI level.

As the edge interface of the IoT ecosystem, the mobile application transforms the standard smartphone into a personalized diagnostic aid and intelligent soft sensor. The operational process, illustrated in Figure 6, displays an actual, unedited system output generated during a live inference trial, showcasing the platform’s ability to process real-world culinary data in real time.

#### 4.1.1 Clinical workflow integration

The user journey initiates when a caregiver or patient captures or uploads a meal image via the primary interface (Figure 6a). The system communicates with the cloud backend to execute the cascaded MLLM pipeline, returning a structured analysis (Figure 6b) that presents detailed nutritional profiles and instance-level IDDSI classifications. A critical safety feature is the customizable patient profile (Figure 6d), which allows caregivers to input a formal clinical prescription (e.g., Level 4 Pureed). This feature ensures that the recommendation engine is strictly bounded by the user’s specific medical requirements, providing a localized and personalized safety audit (Figure 6c) that bridges the gap between hospital-prescribed standards and daily domestic consumption.

### 4.2 Qualitative validation of dish recognition and semantic naming

We validated the initial sensing stage of the platform through qualitative experiments to assess the MLLM’s semantic precision across complex and diverse meal scenes. As summarized in Tables 4–6, the system demonstrates high operational fidelity through three primary logical frameworks.

- Set meal logic. The model successfully resolves complex meal compositions by segmenting a single tray or plate into discrete, manageable components (e.g., rice, grilled chicken, and blanched vegetables). By categorizing these as a set meal, the sensor preserves the texture granularity required for high-resolution safety auditing, effectively preventing the incorrect application of a single IDDSI level to a heterogeneous meal.
- Canonical prioritization. The model prioritizes the identification of specific dishes by their canonical names (e.g., “spaghetti carbonara”). This approach provides a richer semantic prior that allows the system to infer hidden physical hazards—such as fibrous meat textures or mixed-viscosity sauces—leveraging its pre-trained knowledge to anticipate rheological properties before the formal texture audit.
- Visual noise robustness. The sensor effectively filters out non-food artifacts, such as chopsticks, spoons, or tray boundaries, focusing exclusively on culinary items. When exact canonical matches are unavailable, the model utilizes descriptive synthesis to identify ingredients, ensuring that all potential hazards are accounted for within the structured data flow.

**Table 4:** Result visualization of dish recognition from three different inputs of diet images: (a) set meal, (b) single dish (carbonara), and (c) combination of multiple dishes.

Input image	Specific prompt	Output of dishes
(a) 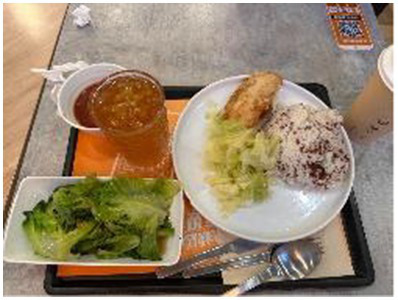	You are an expert culinary vision AI. Analyze this meal image depicting a Hong Kong-style fast food set on a tray, containing a rice bowl, plated cabbage and fried protein, a separate dish of greens, a drink glass, and a sauce container. Segment this meal into distinct items. Output strictly as JSON array: [{“dish_id”: 1, “name”: “…,” “naming_type”: “Canonical OR Descriptive,” “item_type”: “Solid OR Liquid”}]	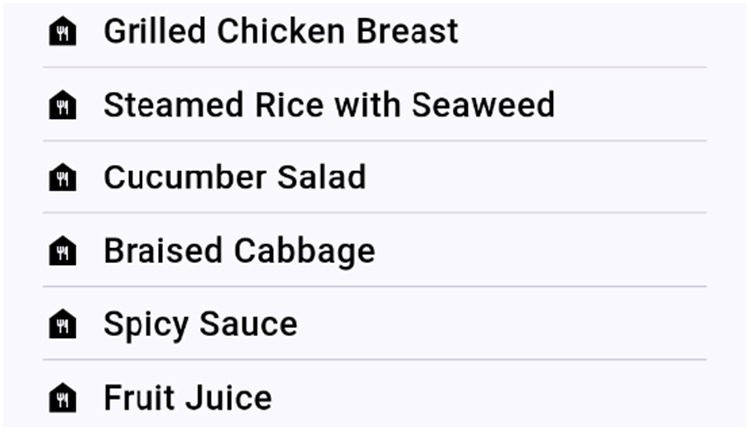
(b) 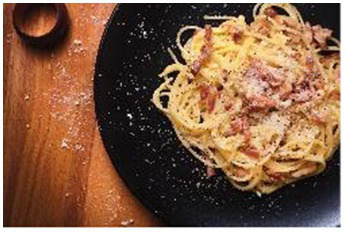	You are an expert culinary vision AI. Analyze this image depicting primarily a single, intricate mixed-texture dish (pasta) served on a black plate, viewed from above. Segment any distinct items (e.g., the main pasta dish, garnishes, or side items if visible), prioritizing canonical names for integrated complex meals. Output strictly as JSON array: [{“dish_id”: 1, “name”: “…,” “naming_type”: “Canonical OR Descriptive,” “item_type”: “Solid OR Liquid”}]	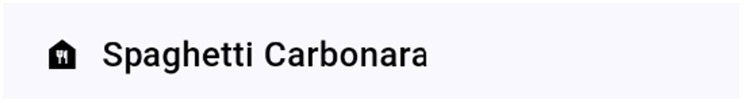
(c) 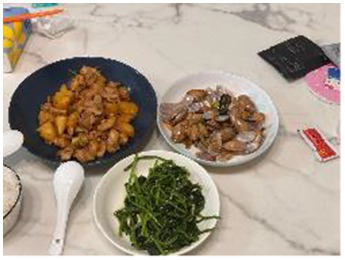	You are an expert culinary vision AI. Analyze this meal image served family style with multiple sharing dishes on a table. Separate the distinct items found on different plates and bowls, including the rice bowl, the leafy greens dish, and the meat dishes, ignoring non-food objects. Output strictly as JSON array: [{“dish_id”: 1, “name”: “…,” “naming_type”: “Canonical OR Descriptive,” “item_type”: “Solid OR Liquid”}]	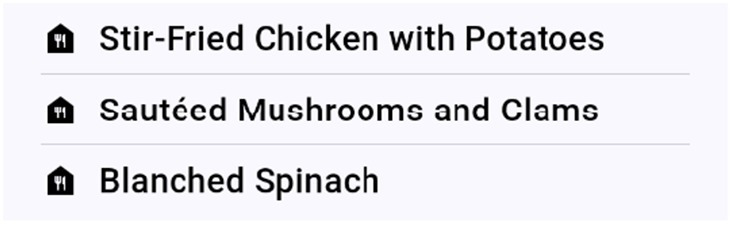

**Table 5:** Result visualization of nutrition retrieval from three different inputs of diet images: (a) set meal, (b) single dish (carbonara), and (c) combination of multiple dishes.

Input image	Detected dishes	Output of nutrition
(a) 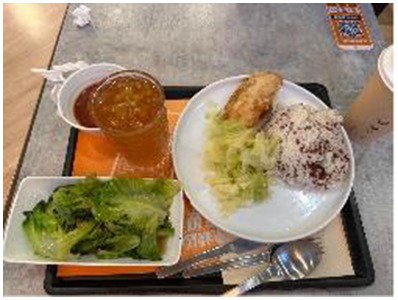	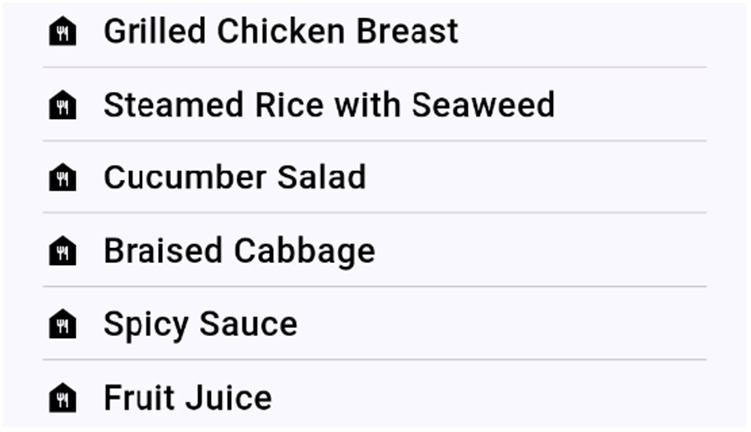	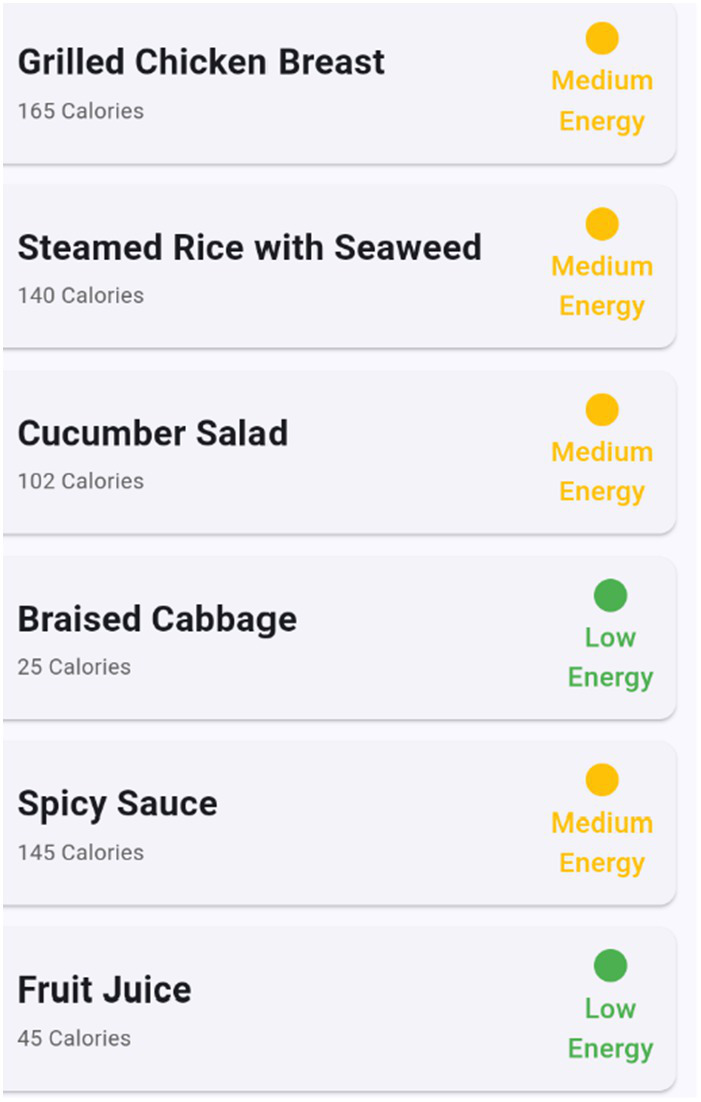
(b) 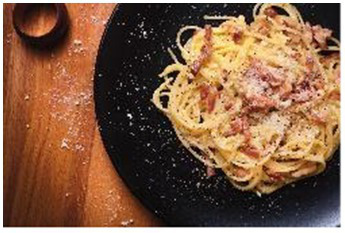	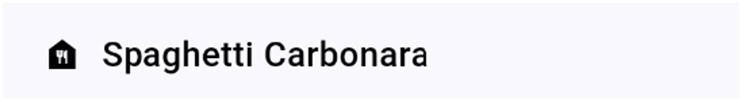	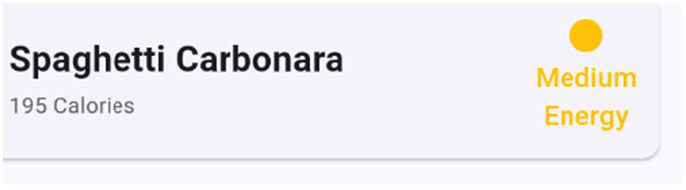
(c) 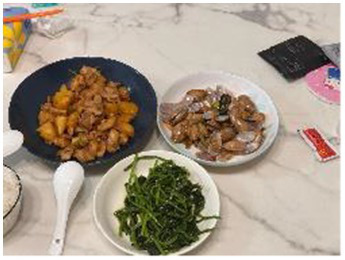	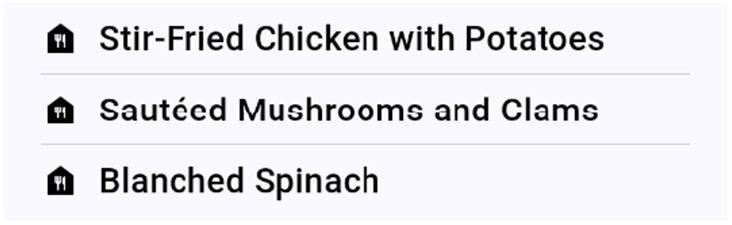	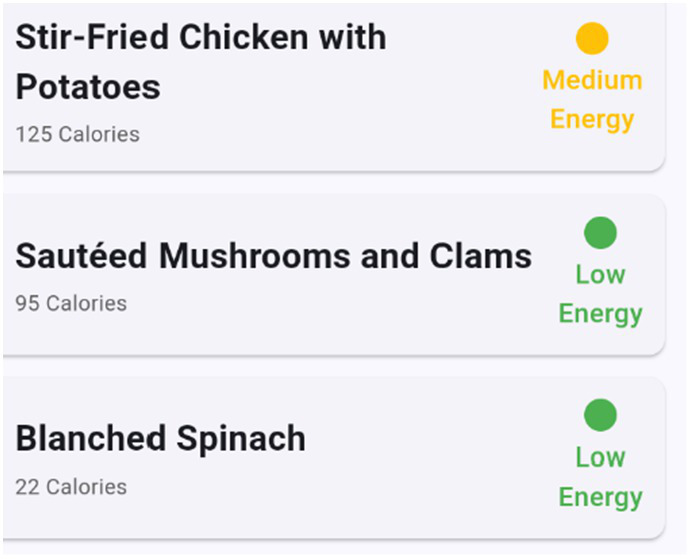

**Table 6:** Result visualization of IDDSI-level classification and suggestion generation from (a) to (c) referring to three different inputs of dish image and (d) referring to the input passed from the preceding naming stage of the pipeline: (a) dish with mixed textures, (b) and (c) similar materials but with different dish information at the IDDSI level, and (d) full output of the dish recognition process.

Input image	Detected dishes	Specific prompt
(a) 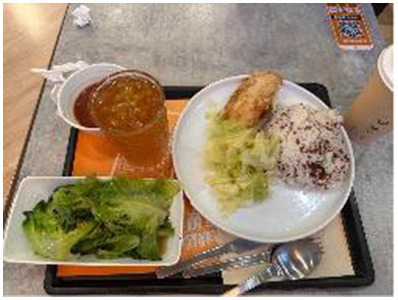	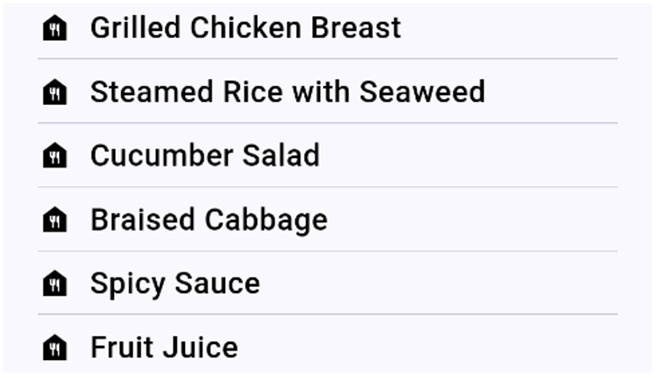	You are an IDDSI specialist. Evaluate the IDDSI safety of the detected dish on the basis of the high-angle visual evidence provided of this meal. Provide a comprehensive audit using IDDSI physical test descriptors (fork pressure, flow simulation) inferred from the image Patient Targets: Solid = Level 4, Liquid = Level 2. Output strictly as JSON array: [{“dish_id”: 1, “name”: “…,” “visual_iddsi_audit”: {…}, “derived_iddsi_level”: 0, “hazard_assessment”: “…,” “modification_suggestion”: “…”}]
Grilled chicken breast, steamed rich with seaweed, cucumber salad → Level 7 Braised cabbage → Level 6 Spicy sauce → Level 3 Fruit juice → Level 0 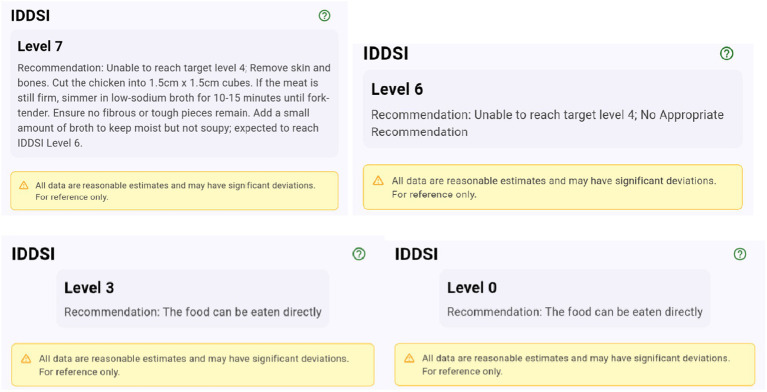		
(b) 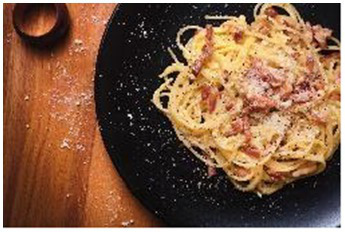	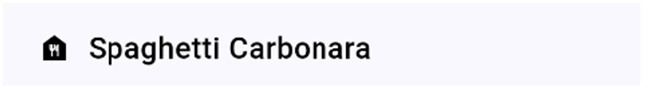	You are an IDDSI specialist. Evaluate the IDDSI safety of the detected dish on the basis of the high-resolution visual evidence provided. Critically assess the inherent dangers of complex, mixed-texture meals Patient Targets: Solid = Level 4, Liquid = Level 2. Output strictly as JSON array: [{“dish_id”: 1, “name”: “…,” “visual_iddsi_audit”: {…}, “derived_iddsi_level”: 0, “hazard_assessment”: “…,” “modification_suggestion”: “…”}]
Spaghetti carbonara → Level 7 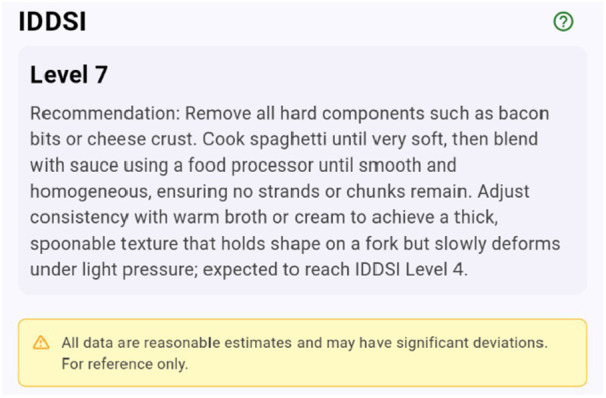		
(c) 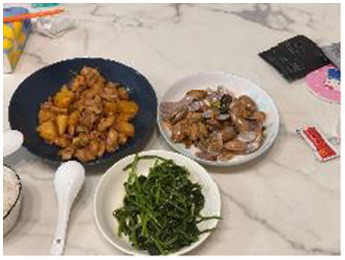	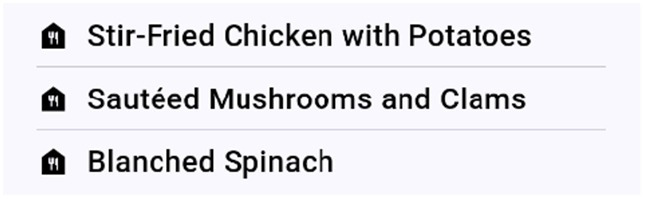	You are an IDDSI specialist. Evaluate the IDDSI safety of the detected dish on the basis of the high-resolution visual evidence provided. Critically assess the inherent dangers of complex, mixed-texture meals Patient Targets: Solid = Level 4, Liquid = Level 2. Output strictly as JSON array: [{“dish_id”: 1, “name”: “…,” “visual_iddsi_audit”: {…}, “derived_iddsi_level”: 0, “hazard_assessment”: “…,” “modification_suggestion”: “…”}]
Stir-fried chicken with potatoes, Sauteed mushrooms, and clams, blanched spinach → Level 7 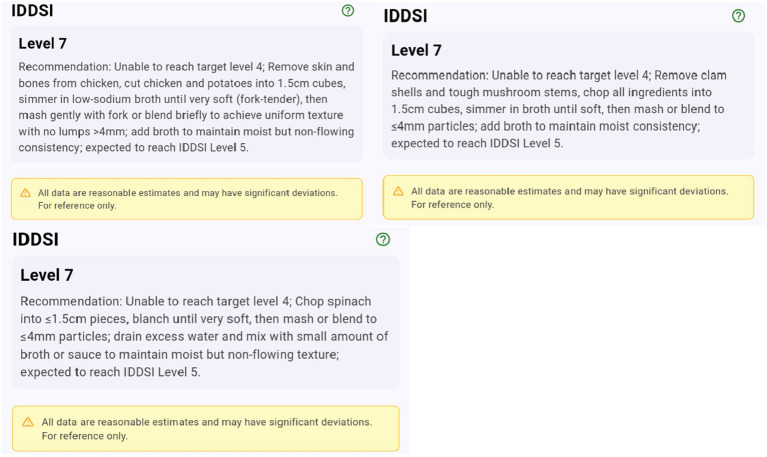		

### 4.3 Benchmark for clinical accuracy: the clinical-expert IDDSI validation benchmark

**Figure 7:**
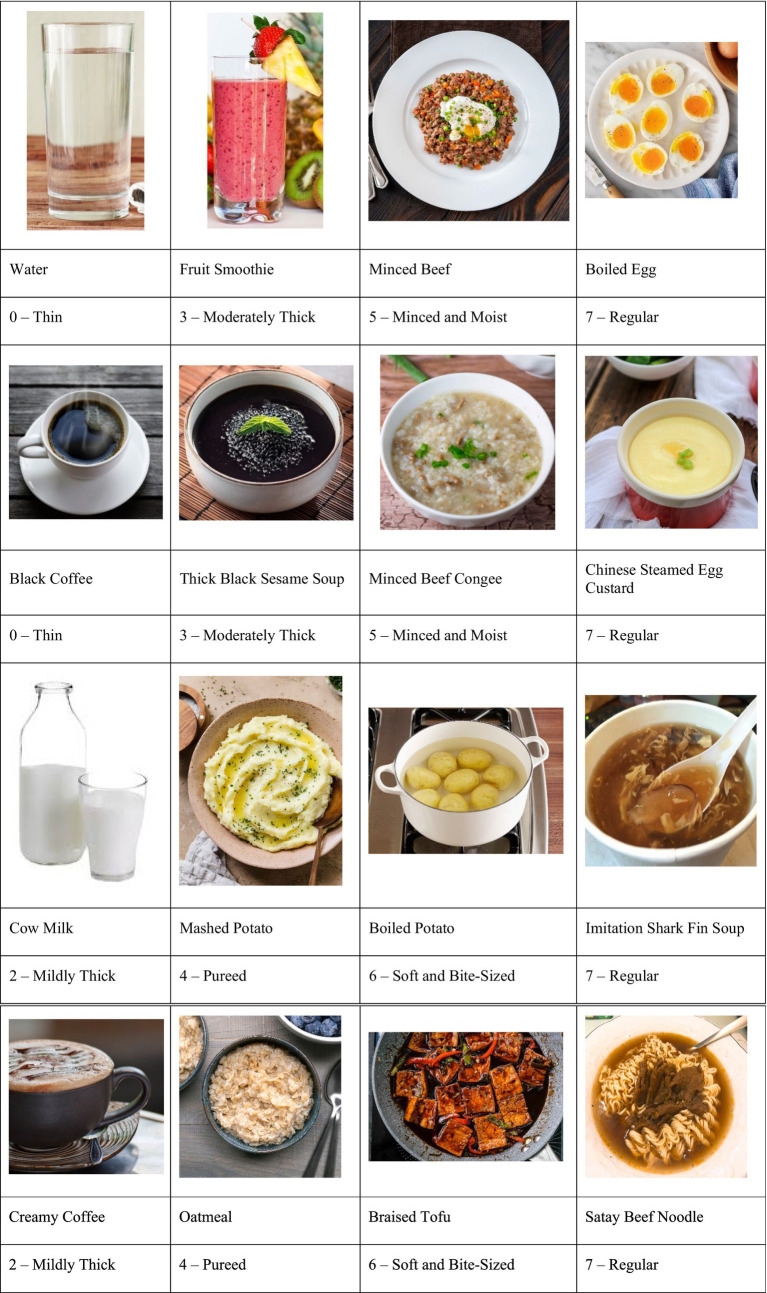
Reference dish images and expert ground-truth IDDSI levels.

To rigorously assess the clinical accuracy and safety of our prototype, we conducted a quantitative validation benchmark verified by certified clinical experts. This process established the Clinical-Expert IDDSI Validation Benchmark (CEIV) Dataset, a specialized repository of 115 diverse meal categories. This benchmark was curated to reflect the unique culinary landscape of Hong Kong, incorporating traditional Cantonese dishes—such as siu mei (BBQ meats) and dim sum—alongside standard Western-style therapeutic meals. Representative visual samples of the meal categories across all eight IDDSI levels are shown in Figure 7.

Dataset provenance and ethical considerations: To ensure complete transparency and reproducibility, the provenance of the 115 meal images constituting the CEIV Benchmark is explicitly declared. The benchmark dataset is constructed as a hybrid image matrix consisting of two distinct subsets: (1) 65 original images capturing local Hong Kong food items (such as dim sum and siu mei) photographed directly by the research team using standard smartphone cameras (Apple iPhone 15), and (2) 50 images representing international and specialized therapeutic textures sourced from online food repositories under open-access Creative Commons (CC BY 4.0) licensing terms. All data collection was conducted or curated to simulate standard domestic kitchen and dining environments under varied consumer-grade ambient lighting conditions, rigorously mirroring real-world home-care deployment landscapes. Because the photographs focus exclusively on isolated dietary plates and food matrices, no identifiable individuals, patient faces, or clinical caregivers appear anywhere within the dataset. Consequently, since the study did not involve human subjects, clinical interventions, or identifiable personal tracking, institutional ethical approval and formal patient consent documentation were not required for this visual asset collection.

#### 4.3.1 Framework universality and cross-cultural transferability

While the current benchmark is anchored in the Hong Kong culinary landscape, the platform’s modular design ensures architectural universality. The cascading MLLM pipeline is decoupled from the underlying nutritional and IDDSI databases, thus making the framework task-agnostic and culturally transferable. Should a user require a different food culture or regional taxonomy, the system can be adapted by simply hot-swapping the retrieval layer. This modularity demonstrates that our proposed intelligent sensing approach is highly flexible for global health applications.

#### 4.3.2 Ground-truth IDDSI level construction

The establishment of a definitive ground truth for this benchmark was overseen by certified clinical experts from the Hospital Authority of Hong Kong. Following official IDDSI Audit Tool protocols (3), each meal was categorized from Level 0 to Level 7 on the basis of specific, measurable physical properties:

- Visual and particle size check: Experts verified that Level 5 (Minced and Moist) samples contained lumps no larger than 4 mm and Level 6 (Soft and Bite-Sized) pieces did not exceed 15 mm × 15 mm.
- The IDDSI flow test: For liquid and transitional categories (Levels 0–4), the standard 10 mL syringe flow test was utilized to confirm precise viscosity levels on the basis of the volume of liquid remaining after 10 s of flow.
- Mechanical testing: Experts performed fork pressure tests to evaluate hardness. Level 5 textures were required to be mashable with pressure that does not cause the thumbnail to blanch (turn white), whereas Level 6 textures required sufficient pressure to turn the thumbnail white.
- Spoon tilt test: For Level 4 (Pureed) and Level 5, samples held their shape on a spoon and slid off cleanly when tilted, leaving minimal residue—confirming appropriate cohesiveness and the absence of stickiness.

This rigorous validation process ensures that the MLLM results are compared against a medically sound baseline, providing a reliable measure of the system’s performance as a safety-critical screening tool. Representative samples of the CEIV Benchmark are documented in Table 7.

**Table 7:** Expert-verified IDDSI benchmark samples.

Food item	IDDSI level	Remarks/caution	Item type
Water	0—Thin	Risk of aspiration; consider thickener if indicated	Liquid
Black coffee	0—Thin	Risk of aspiration; consider thickener if indicated	Liquid
Cow milk	2—Mildly thick	Check consistency; varies by product	Liquid
Creamy coffee	2—Mildly thick	Check consistency; varies by product	Liquid
Fruit smoothie	3—Moderately thick	Drinkable but not thin	Liquid
Thick black sesame soup	3—Moderately thick	Drinkable but not thin	Liquid
Mashed potato	4—Pureed	No chewing required; must be smooth	Solid
Oatmeal	4—Pureed	No chewing required; must be smooth	Solid
Minced beef	5—Minced and moist	Pieces ≤4 mm; moist only	Solid
Minced beef congee	5—Minced and moist	Pieces ≤4 mm; moist only	Solid
Boiled potato	6—Soft and bite-sized	Pieces ≤15 mm; tender	Solid
Braised tofu	6—Soft and bite-sized	Pieces ≤15 mm; tender	Solid
Boiled egg	7—Regular	May require modification for dysphagia	Solid
Chinese steamed egg custard	7—Regular	May require modification for dysphagia	Solid
Imitation shark fin soup	7—Regular	Mixed texture; high choking risk	Solid
Satay beef noodle	7—Regular	Mixed texture; high choking risk	Solid

### 4.4 Comparative model performance and clinical metrics

The performance of the integrated MLLM was benchmarked against leading frontier models, including Gemini, ChatGPT, and DeepSeek (Table 8). The models were evaluated using five primary metrics designed to capture analytical precision and patient safety to ensure the assessment was clinically relevant for a digital healthcare environment:

- Exact match accuracy (EMA): This measures the frequency with which the model identifies the precise, expert-validated IDDSI level. It serves as the baseline for the intelligent sensor’s overall reliability.
- Adjacent accuracy (±1 Level): This tracks the rate at which the model predicts a level within one increment of the expert judgment. In clinical nutrition, this indicates a reliable understanding of the texture “neighborhood,” where minor deviations are often less critical than major category jumps.
- Mean absolute error (MAE): The average numerical deviation from the ground truth. This provides insight into the magnitude of misclassifications; a low MAE indicates that even when the model is wrong, it remains close to the safety threshold.
- Critical safety error rate (CSER): This is a high-stakes safety metric monitoring “optimistic” misclassifications, where the model predicts a texture to be two or more levels lower (softer or thinner) than the expert ground truth. Such errors are flagged as high risk, as they could lead to a caregiver providing unsafe “regular” textures to a patient restricted to pureed or minced diets.
- Bi-directional FNR: The primary safety metric monitoring missed hazards. In our framework, a false negative occurs when a hazard is present but not detected. Given the inverse risk profiles of dysphagia, bi-directional FNR is calculated bi-directionally:

1. Solid hazard FNR: Underestimating the hardness of a solid (predicting a lower IDDSI level).
2. Liquid hazard FNR: Overestimating the viscosity of a liquid (predicting a higher IDDSI level than the actual thin consistency).

**Table 8:** Model registry: comparison of LLMs and checkpoint metadata.

Model	LLM category	Source of LLMs	Date of access/creation of checkpoints
DeepSeek	Web-based commercial LLMs	https://chat.deepseek.com/	28/04/2026
ChatGPT	Web-based commercial LLMs	https://chat.openai.com/	28/04/2026
Gemini	Web-based commercial LLMs	https://gemini.google.com/	28/04/2026
Qwen (used in our platform)	Web-based commercial LLMs	https://dashscope-intl.aliyuncs.com/api	30/04/2026
GPT5.4_mini	Commercial LLMs (Snapshots)	University internal platform	28/04/2026
GPT4.1	Commercial LLMs (Snapshots)	University internal platform	28/04/2026
Grok4.1	Commercial LLMs (Snapshots)	University internal platform	28/04/2026
Llama3.3	Commercial LLMs (Snapshots)	University internal platform	28/04/2026
DeepSeek-v3.2	Commercial LLMs (Snapshots)	University internal platform	28/04/2026
Mistral-Small-3.2-24B-Instruct	Hugging face checkpoint	Transformers library V5.3.0	06/06/2025
Gemma3-27B	Hugging face checkpoint	Transformers Library V5.3.0	04/04/2025
aya-vision-32b	Hugging face checkpoint	Transformers Library V5.3.0	04/04/2025
medgemma-27b-it	Hugging face checkpoint	Transformers Library V5.3.0	04/06/2025
LFM2-VL-3B	Hugging Face Checkpoint	Transformers Library V5.3.0	10/10/2025

This validation framework ensures that the prototype’s suggestions—such as modifying “Regular” BBQ pork into “Minced and Moist” by finely dicing it to <4 mm—are not only culturally relevant but also clinically sound.

### 4.5 Analysis of clinical viability

The comparative performance across all tested models, including EMA and CSER, is presented in Table 9. The quantitative results reveal a critical safety advantage for the Qwen API-driven pipeline, which achieved the lowest total FNR of 9.20%. A key observation is the evident decoupling of raw exact-match accuracy from actual clinical safety. Models such as GPT 4.1 exhibited higher raw accuracy (70.43% EMA) yet concurrently failed to identify nearly 20% of critical textural hazards. In the context of dysphagia care—where a single missed hazard can lead to a life-threatening aspiration or choking event—our findings suggest that a model’s safety bias (minimized FNR) is a more significant metric for intelligent soft sensors than its raw analytical accuracy.

**Table 9:** Quantitative evaluation: clinical accuracy metrics.

Model	EMA (Mean ± STD, %)	Adj Acc (Mean ± STD, %)	MAE (Mean ± STD)	CSER (Mean ± STD, %)
Web-based commercial LLMs (most recently updated)				
DeepSeek	66.96 ± 47.24	81.74 ± 38.80	0.870 ± 1.699	18.26 ± 38.80
ChatGPT	64.35 ± 48.11	81.74 ± 38.80	0.878 ± 1.676	17.39 ± 38.07
Gemini	62.61 ± 48.60	80.00 ± 40.18	0.904 ± 1.665	15.65 ± 36.49
Qwen (used in our platform)	61.74 ± 48.82	80.00 ± 40.18	0.809 ± 1.395	12.17 ± 32.84
Commercial LLMs (Snapshots)				
GPT5.4_mini	61.74 ± 48.82	80.00 ± 40.18	0.922 ± 1.666	20.00 ± 40.18
GPT4.1	70.43 ± 45.83	80.87 ± 39.50	0.826 ± 1.682	19.13 ± 39.50
Grok4.1	65.22 ± 47.84	81.74 ± 38.80	0.878 ± 1.676	17.39 ± 38.07
Llama3.3	52.17 ± 50.17	78.26 ± 41.43	1.035 ± 1.691	19.13 ± 39.50
DeepSeek-v3.2	49.57 ± 50.22	74.78 ± 43.62	1.191 ± 1.825	25.22 ± 43.62
Hugging face checkpoint inference				
Mistral-Small-3.2-24B-Instruct	44.35 ± 49.90	71.30 ± 45.43	1.113 ± 1.296	10.43 ± 30.70
Gemma3-27B	39.13 ± 49.02	75.65 ± 43.11	1.191 ± 1.444	9.57 ± 29.54
aya-vision-32b	32.17 ± 46.92	66.09 ± 47.55	1.400 ± 1.456	20.87 ± 40.82
medgemma-27b-it	20.00 ± 40.18	68.70 ± 46.58	1.374 ± 1.120	12.17 ± 32.84
LFM2-VL-3B	11.30 ± 31.80	33.04 ± 47.24	2.113 ± 1.190	50.43 ± 50.22

#### 4.5.1 The liquid failure mode

The comparative performance across all tested models of FNR divided into solid and liquid is presented in Table 10. A significant finding is the categorical failure of localized, open-source models—specifically Mistral-Small, Gemma3-27B, and aya-vision—to accurately interpret liquid rheology. All tested localized checkpoints exhibited a 100% liquid FNR, effectively “hallucinating” safety for every liquid aspiration risk encountered in the benchmark. This failure mode suggests that current localized architectures may lack the high-dimensional visual reasoning required to differentiate between subtle optical markers of thin versus thick liquid flow. By contrast, our cloud-orchestrated pipeline maintained a robust 7.14% liquid FNR, demonstrating that the high-capacity reasoning of frontier MLLMs is currently a fundamental requirement for medical-grade liquid monitoring in digital healthcare.

**Table 10:** Quantitative evaluation: clinical safety (FNR) by food phase divided into solid (4–7) and liquid (0–3).

Model	FNR (Mean ± STD, %)	Solid FNR (Mean ± STD, %)	Liquid FNR (Mean ± STD, %)	EMA (Mean ± STD, %)	Solid EMA (Mean ± STD, %)	Liquid EMA (Mean ± STD, %)
Web-based commercial LLMs (most recently updated)						
DeepSeek	19.54 ± 39.88	23.29 ± 42.56	0 ± 0.00	66.96 ± 47.24	65.88 ± 47.69	70.00 ± 46.61
ChatGPT	19.54 ± 39.88	23.29 ± 42.56	0 ± 0.00	64.35 ± 48.11	62.35 ± 48.74	70.00 ± 46.61
Gemini	19.54 ± 39.88	19.18 ± 39.64	21.43 ± 42.58	62.61 ± 48.60	68.24 ± 46.83	46.67 ± 50.74
Qwen (used in our platform)	9.20 ± 29.06	9.59 ± 29.65	7.14 ± 26.73	61.74 ± 48.82	63.53 ± 48.42	56.67 ± 50.40
Commercial LLMs (Snapshots)						
GPT5.4_mini	20.69 ± 40.74	24.66 ± 43.40	0 ± 0.00	61.74 ± 48.82	60.00 ± 49.28	66.67 ± 47.95
GPT4.1	19.54 ± 39.88	23.29 ± 42.56	0 ± 0.00	70.43 ± 45.83	75.29 ± 43.39	56.67 ± 50.40
Grok4.1	19.54 ± 39.88	23.29 ± 42.56	0 ± 0.00	65.22 ± 47.84	67.06 ± 47.28	60.00 ± 49.83
Llama3.3	14.94 ± 35.86	17.81 ± 38.52	0 ± 0.00	52.17 ± 50.17	52.94 ± 50.21	50.00 ± 50.85
DeepSeek-v3.2	21.84 ± 41.55	26.03 ± 44.18	0 ± 0.00	49.57 ± 50.22	43.53 ± 49.87	66.67 ± 47.95
Hugging face checkpoint inference						
Mistral-Small-3.2-24B-Instruct	25.29 ± 43.72	10.96 ± 31.45	100.00 ± 0.00	44.35 ± 49.90	56.47 ± 49.87	10.00 ± 30.51
Gemma3-27B	26.44 ± 44.36	12.33 ± 33.10	100.00 ± 0.00	39.13 ± 49.02	50.59 ± 50.29	6.67 ± 25.37
aya-vision-32b	35.63 ± 48.17	23.29 ± 42.56	100.00 ± 0.00	32.17 ± 46.92	42.35 ± 49.71	3.33 ± 18.26
medgemma-27b-it	26.44 ± 44.36	13.70 ± 34.62	92.86 ± 26.73	20.00 ± 40.18	25.88 ± 44.06	3.33 ± 18.26
LFM2-VL-3B	85.06 ± 35.86	82.19 ± 38.52	100.00 ± 0.00	11.30 ± 31.80	14.12 ± 35.03	3.33 ± 18.26

### 4.6 Technical configuration and temperature sensitivity

To ensure the clinical reliability and reproducibility of the intelligent soft sensor, we established a standardized inference environment for all benchmarked models.

#### 4.6.1 Deterministic baseline

For the CEIV benchmark, all models were configured to operate in a deterministic state. For API-integrated models (e.g., Qwen-VL-Max and Gemini 3), the temperature was set to its absolute minimum (*T* = 0.01). For localized Hugging Face checkpoints, we implemented greedy decoding by setting do_sample = False. This configuration is a prerequisite for medical-grade monitoring, ensuring that the same meal image consistently yields the same IDDSI classification and nutritional profile, thereby eliminating the safety risks associated with stochastic “hallucinations.”

#### 4.6.2 Temperature sensitivity analysis

To investigate the model’s robustness under varying degrees of “creative” reasoning, we conducted a sensitivity analysis across four temperature tiers (*T* = 0.01, 0.3, 0.7, and 1.0) as documented in Table 11.

- Impact on safety (FNR): Our results indicate that as temperature increases, the total FNR significantly deteriorates. For instance, Qwen-VL-Max maintained a 9.2% FNR at *T* = 0.01 but showed increased variance and missed hazards at *T* = 0.7, where the stochastic selection of tokens often bypassed the conservative safety priors established in our prompt engineering scaffolding.
- Logical consistency: Higher temperatures (T > 0.7) disrupt the CoT reasoning chain. The model occasionally skipped critical fork pressure simulation steps, jumping directly to a final IDDSI assignment without verifying surface pattern retention.

**Table 11:** Temperature sensitivity analysis on Qwen API and Gemma3-27B.

(a) Clinical accuracy metrics				
Temperature	EMA (Mean ± STD, %)	Adj Acc (Mean ± STD, %)	MAE (Mean ± STD)	CSER (Mean ± STD, %)
Qwen (used in our platform)				
0.01	61.74 ± 48.82	80.00 ± 40.18	0.852 ± 1.535	10.43 ± 30.70
0.3	57.39 ± 49.67	80.87 ± 39.50	0.896 ± 1.530	11.30 ± 31.80
0.7	60.87 ± 49.02	81.74 ± 38.80	0.826 ± 1.446	8.70 ± 28.30
1.0	58.26 ± 49.53	77.39 ± 42.01	0.983 ± 1.638	13.91 ± 34.76
Hugging face checkpoint (Gemma3-27B)				
0.01	42.61 ± 49.67	72.17 ± 45.01	1.157 ± 1.399	11.30 ± 31.80
0.3	42.61 ± 49.67	72.17 ± 45.01	1.157 ± 1.399	11.30 ± 31.80
0.7	41.74 ± 49.53	72.17 ± 45.01	1.174 ± 1.403	12.17 ± 32.84
1.0	41.74 ± 49.53	71.30 ± 45.01	1.148 ± 1.306	11.30 ± 31.80

(b) Clinical safety (FNR) by food phase divided into solid (4–7) and liquid (0–3)						
Temperature	FNR (Mean ± STD, %)	Solid FNR (Mean ± STD, %)	Liquid FNR (Mean ± STD, %)	EMA (Mean ± STD, %)	Solid EMA (Mean ± STD, %)	Liquid EMA (Mean ± STD, %)
Qwen (used in our platform)						
0.01	9.20 ± 29.06	8.22 ± 27.66	7.14 ± 26.73	61.74 ± 48.82	68.24 ± 46.83	43.33 ± 50.40
0.3	10.34 ± 30.63	9.59 ± 29.65	14.29 ± 36.31	57.39 ± 49.67	61.18 ± 49.02	46.67 ± 50.74
0.7	10.05 ± 27.36	8.22 ± 27.66	14.29 ± 36.31	60.87 ± 49.02	63.53 ± 48.42	53.33 ± 50.74
1.0	12.64 ± 33.43	10.96 ± 31.45	21.43 ± 42.58	58.26 ± 49.53	63.53 ± 48.42	43.33 ± 50.40
Hugging face checkpoint (Gemma3-27B)						
0.01 (do-sample = False)	27.59 ± 44.95	13.70 ± 34.62	100.00 ± 0.00	42.61 ± 49.67	57.65 ± 49.71	0 ± 0.00
0.3	28.74 ± 45.52	15.07 ± 36.02	100.00 ± 0.00	41.74 ± 49.53	55.29 ± 50.01	3.33 ± 18.26
0.7	28.74 ± 45.52	15.07 ± 36.02	100.00 ± 0.00	41.74 ± 49.53	55.29 ± 50.01	3.33 ± 18.26
1.0	27.59 ± 44.95	13.70 ± 34.62	100.00 ± 0.00	42.61 ± 49.67	57.65 ± 49.71	0 ± 0.00

Consequently, we conclude that a low-temperature regime (*T* = 0, and *T* = 0.01 if it could not be set to 0) is not merely a preference but a safety-critical requirement for MLLM-based dysphagia auditing.

### 4.7 System latency and resource efficiency

To demonstrate the operational viability of the platform in real-world clinical or home-care settings, we report on system latency and token consumption across meals of varying complexity. To establish a rigorous baseline for consistency and address previous evaluation gaps, all performance metrics are expressed as mean values accompanied by their sample standard deviations (±std) gathered over repeated 5 inference trials. Detailed step-wise performance metrics are provided in Table 12, while the relationship between meal complexity and resource scaling is documented in Table 13.

**Table 12:** Computational efficiency: step-wise latency and token consumption.

Test image: 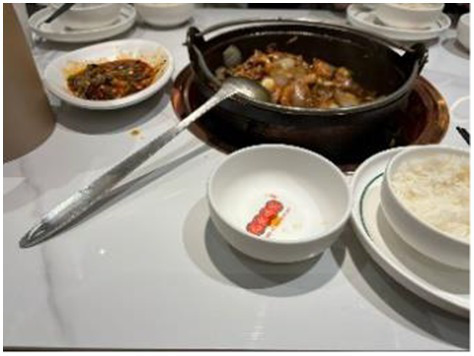	Time (Mean ± STD, s)	Input token (Mean ± STD)	Output token (Mean ± STD)
Qwen (used in our platform)			
Step 1: Dish recognition	5.93 ± 2.86	1,299 ± 0	98 ± 1
Step 2: Nutrition retrieval (From CFS database)	1.12 ± 0.12	/	/
Step 3: IDDSI classification and suggestion generation	18.70 ± 3.01	1,431 ± 1	615 ± 41
Total	25.75 ± 4.13	2,730 ± 1	713 ± 21
Web-based online platform (Gemini)			
Step 1: Dish recognition	/	/	/
Step 2: Nutrition retrieval	/	/	/
Step 3: IDDSI classification and suggestion generation	/	/	/
Total (No statistics for each step)	21.72 ± 8.47	3,123 ± 35	10,600 ± 101
Hugging face checkpoint (Gemma3-27B)			
Step 1: Dish recognition	21.36 ± 0.98	328 ± 0	211 ± 0
Step 2: Nutrition retrieval	1.34 ± 0.02	/	/
Step 3: IDDSI classification and suggestion generation	101.95 ± 0.01	579 ± 0	1,011 ± 0
Total	124.65 ± 0.98	907 ± 0	1,222 ± 0

All the values are presented in Mean ± STD in 5 trials.

**Table 13:** Operational stability: meal complexity vs. resource usage.

Complexity	Meal items	Total latency time (Mean ± STD, s)	Total tokens (input/output) (Mean ± STD)	Average time per meal (Mean ± STD, s)	Average token per meal (Mean ± STD, s)
Qwen (used in our platform)					
Low complexity	1	27.78 ± 2.69	1,250 ± 1,084	27.78 ± 2.69	1,250 ± 1,084
Low complexity	3	37.25 ± 8.72	8,886 ± 1,000	12.41 ± 3.66	2,962 ± 876
Middle complexity	5	65.09 ± 23.20	12,417 ± 822	13.01 ± 2.87	2,483 ± 384
High complexity	7	84.33 ± 10.33	13,578 ± 3,303	12.04 ± 5.07	1939 ± 373
High complexity	9	102.04 ± 12.19	18,605 ± 2,459	11.33 ± 5.48	2067 ± 294
Hugging face checkpoint (Gemma3-27B)					
Low complexity	1	48.30 ± 21.31	1,381 ± 527	48.30 ± 21.31	1,381 ± 527
Low complexity	3	106.83 ± 11.56	3,117 ± 118	35.61 ± 6.85	1,039 ± 156
Middle complexity	5	150.97 ± 14.26	4,730 ± 141	30.20 ± 22.09	946 ± 80
High complexity	7	189.15 ± 1.10	6,220 ± 33	27.02 ± 1.74	888 ± 30
High complexity	9	207.99 ± 4.17	7,567 ± 41	23.11 ± 3.61	841 ± 49

#### 4.7.1 HPC inference environment for local models

To establish a baseline for offline deployment and assess the requirements for edge-based clinical monitoring, local inference for the Gemma 3-27B checkpoint was conducted on a high-performance computing (HPC) cluster. The hardware environment consisted of 2* NVIDIA H20 (40 GB VRAM). The inference pipeline utilized the PyTorch 2.3 framework with Transformers Library V5.3.0 for high-throughput processing.

#### 4.7.2 Step-wise performance analysis

An analysis of the three-stage pipeline highlights the trade-offs between cloud-orchestrated reasoning and localized inference. The Qwen API achieved a total processing time of 25.75 ± 4.13 s for the benchmark meal, with Step 3 (safety audit) being the primary computational bottleneck at 18.70 ± 3.01 s because of the extensive CoT reasoning required to simulate physical tests. By contrast, the web-based Gemini platform, while reporting a slightly faster total pipeline mean of 21.72 s, exhibited substantial network-induced timing fluctuations (±8.47 s) and produced a staggering 10,600 ± 101 output tokens. Our structured pipeline achieved equivalent clinical precision with only 713 ± 21 tokens, representing a 93% reduction in output overhead. Despite HPC acceleration, the local Gemma 3-27B required 124.65 ± 0.98 s (comprising 21.36 ± 0.98 s for recognition, 1.34 ± 0.02 s for nutrition retrieval, and 101.95 ± 0.01 s for IDDSI level prediction), indicating that high-parameter models still face significant latency gaps for real-time edge use.

#### 4.7.3 Operational stability and complexity scaling

The system demonstrates the high efficiency, where the marginal computational cost per meal item decreases as the visual scene becomes more complex. For the Qwen API, a single-item meal requires 27.78 s, but a high-complexity meal of nine distinct items was processed in 102.04 s. The average processing time per meal item is thus reduced to just 11.33 s, a 59% reduction in per-item latency. Similarly, average token consumption per item exhibits a downward trend as complexity increases, dropping from 1,250 tokens for a single dish to approximately 2,067 tokens for a nine-item configuration. This result suggests that the MLLM efficiently shares global visual context across segmented localizations.

#### 4.7.4 Infrastructure error tolerance and telemetry

To ground the system’s operational reliability under maximum load constraints, the pipeline was subjected to a high-frequency stress test of 100 continuous end-to-end dispatches, establishing the provider’s practical API request frequency ceiling. The result is shown in Table 14. Telemetry logs revealed an initial network timeout rate (HTTP 504) of 2.0% (*N* = 2) caused by transient server-side socket saturation, alongside a localized rate-limiting constraint (HTTP 429) of 2.0% (*N* = 2) encountered automatically after the 89th consecutive dispatch. Crucially, the platform’s integrated 3-tier error-handling architecture natively intercepted both infrastructure anomalies. By deploying automated exponential back-off delays, the system bypassed rate-limit windows and resolved network drops without session termination or user-end application crashes. This orchestration achieved a critical post-retry failure rate of 0.0% (*N* = 0), driving a 100.0% final request completion rate.

**Table 14:** API operational stability and failure statistics (100 continuous trials).

Operational stability metric	Quantitative value	System status
Test image	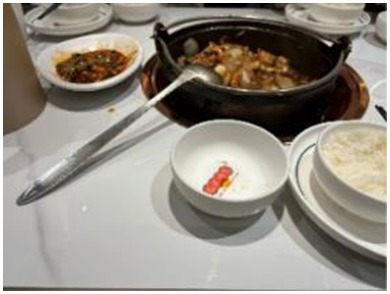	Same as the test image of Table 12
Total inference requests dispatched	100	Upper limit of Qwen API request frequency in short time
Initial network timeouts (HTTP 504)	2.0% (*N* = 2)	Intercepted by 3-tier back-off logic
Transient rate limiting (HTTP 429)	2.0% (*N* = 2)	Triggered after request 89/100; resolved via automated back-off delay
Critical API failures (post-retry)	0.0% (*N* = 0)	Fully mitigated
Final request completion rate	100.0%	Operationally stable

#### 4.7.5 Clinical deployment feasibility

These metrics confirm that the platform is a suitable near-real-time clinical proxy tool. By utilizing cloud-orchestrated sensing, the system provides accurate texture predictions and modification suggestions within a clinically acceptable time frame—typically under 30 s for standard meals—without necessitating high-end edge hardware at the point of consumption. This efficiency ensures that the intelligent soft sensor remains accessible via standard consumer smartphones, providing reliable oversight and safety guidance in diverse care settings.

### 4.8 Visual differentiation logic for liquids vs. solids

A critical component of the system’s clinical safety profile is its ability to distinguish between Level 3 (liquidized/moderately thick) and Level 4 (pureed/extremely thick) textures. These levels represent a high-stakes intersection in the IDDSI framework, as Level 3 belongs to the “drink” pyramid while Level 4 is the bridge between “food” and “drinks.” The comprehensive list of expert-verified food items and their clinical texture hazard remarks can be found in Table 15.

**Table 15:** Qualitative visual markers for liquid IDDSI phase differentiation.

IDDSI level	Rheological property	Visual/optical marker	Clinical audit counterpart
Level 0 (Thin)	Low viscosity, rapid flow	Flat meniscus, high transparency at edges, rapid splashing or ripples	10 ml syringe test (leaves <1 mL)
Level 2 (Mildly thick)	Mild viscosity, delayed flow	Slight surface tension, leaves a thin, translucent coating on the sides of a cup or spoon	10 ml syringe test (leaves 4–8 mL)
Level 3 (Moderately thick)	High viscosity, self-leveling	Slow gravitational drip from fork prongs (dollops), self-levels evenly in a container, leaves a thick, opaque coating	Fork drip test/syringe test
Level 4 (Pureed)	Solid-like phase, no flow	Surface pattern retention (embossed fork marks remain visible), sits in a stable mound, does not self-level	Spoon tilt test/fork pressure

#### 4.8.1 Visual reasoning cues for phase differentiation

The MLLM utilizes specific visual boundary markers derived from the IDDSI Audit Tool to differentiate fluid samples from pureed solids:

- Surface pattern retention: The model identifies Level 4 pureed solids by their ability to maintain a clear, embossed pattern on the surface (e.g., marks left by a fork or spoon). Conversely, Level 3 liquidized samples are identified by a lack of pattern retention; the MLLM infers that the substance’s viscosity is low enough to cause self-leveling under gravity.
- Gravitational behavior (fork drip simulation): The system analyzes the interaction between the substance and fork prongs. Level 3 liquids drip slowly and continuously through the prongs in distinct dollops. By contrast, Level 4 solids sit in a stable mound above the prongs, with only a small “tail” potentially forming below, indicating high cohesiveness.
- Cohesion and adhesion (spoon tilt simulation): While Level 3 pours easily from a spoon when tilted, Level 4 holds its shape on a teaspoon even when inverted or tilted. The model looks for the “sliding” characteristic—where the bolus releases as a single unit with minimal residue—to confirm safety.

#### 4.8.2 Recommendation pathway divergence

Once a liquid-phase or transitional substance (IDDSI Levels 0–3) is detected, the pipeline shifts its recommendation logic:

- Rheological vs. mechanical modification: For solid foods (Levels 5–7), the system suggests mechanical resizing, such as dicing to <4 mm for Level 5 or <15 mm for Level 6. When a liquid is detected, the logic bypasses particle size reduction and shifts to viscosity management.
- Standardized flow recommendations: The pathway for liquids prioritizes the IDDSI Flow Test. Recommendations change from chopping to thickening, with the MLLM suggesting specific commercial thickening agent ratios or starch-based additives to ensure the fluid meets the 10-s syringe flow requirements for the target level (e.g., ensuring 8–10 mL remains in the syringe for Level 3).
- Management of “thin” separation: If the visual input reveals a mixed-texture food where juice or thin liquid separates from the solid (e.g., tinned fruit), then the system identifies the food as a high-risk Level 7 texture. The recommendation logic then directs the user to strain the thin liquid or puree the entire dish to Level 4 to eliminate aspiration risk.

## 5 Discussion

The experimental results demonstrate that MLLMs can achieve high clinical alignment with IDDSI standards, particularly when the underlying architecture is optimized for safety-first performance. However, transitioning from a technical feasibility prototype to a robust bedside clinical tool requires a nuanced understanding of its operational boundaries, its long-term clinical implications, and the inherent modality–construct gap, which is the fundamental challenge of inferring mechanical physics (rheological and textural properties) from two-dimensional computer vision (optical data).

### 5.1 Clinical implications: closing the texture–safety gap

The primary clinical value of this framework is that it functions as a point-of-consumption safety audit. While clinical experts provide precise dietary prescriptions (e.g., IDDSI Level 4), a significant texture–safety gap often emerges once patients are discharged to home care. Caregivers frequently struggle to visually verify if a prepared meal meets these mechanical requirements, leading to accidental aspiration.

By providing a real-time second opinion, our platform reduces the cognitive load on caregivers and empowers them with a clinical proxy tool. Furthermore, the IoT-based logging allows for long-term adherence monitoring, providing clinicians with data-driven insights into dietary compliance outside the hospital environment.

### 5.2 Deployment constraints and operational realities

Even though the platform is a promising automated diagnostic aid, it has several real-world operational constraints that must be acknowledged to guide future large-scale deployment.

#### 5.2.1 Hardware variability and optical fidelity

Clinical performance is intrinsically linked to the quality of the input data. Hardware variability—specifically regarding camera sensor resolution and lens distortion—remains a significant factor. In home environments, suboptimal lighting conditions or low-resolution sensors can obscure critical visual markers, such as surface granularity or liquid meniscus behavior, which are essential for accurate IDDSI classification. Future iterations may require standardized calibration steps or automated image-quality checks before the inference pipeline initiates to maintain the reliability of the intelligent soft sensor.

#### 5.2.2 Nutritional reference abstraction and portion mass bounds

A primary constraint of the current implementation is located within the nutritional tracking layer. Because the system provides static per-100 g reference database lookups from the CFS repository instead of calculating actual portion mass or 3D thickness profiles, absolute nutritional values carry an unquantified error proportional to the user’s portion variations. While this is acceptable for tracking relative longitudinal dietary choices and enforcing baseline component identification, clinicians and caregivers must note that it does not replace precise, physical gravimetric portion weighing.

#### 5.2.3 User-end response time (URT) and connectivity

The URT is a critical factor for clinical adoption. While our cloud-based pipeline is highly efficient for a pre-meal check, the inherent network latency suggests the system is currently best positioned as a screening tool rather than a real-time active monitoring device during the mechanical act of deglutition.

#### 5.2.4 Path toward offline resilience

Future iterations that utilize optimized, quantified local models—such as Qwen3-VL or Qwen3.5—are necessary to address the limitations of network dependency. Moving the inference logic to the edge will facilitate offline, low-latency deployment in connectivity-limited environments, such as rural care facilities or transit settings. Doing so will ensure that the safety-critical audit remains accessible regardless of infrastructure stability, further democratizing medical-grade texture monitoring.

### 5.3 The “texture proxy” boundary: screening vs. diagnosis

A fundamental distinction must be maintained regarding the clinical scope of this technology: This system evaluates the food, not the patient. Our framework functions as a texture screening proxy that identifies if a specific meal presents a mechanical hazard relative to a pre-existing clinical prescription. It is not intended to, nor can it, replace gold-standard diagnostic procedures such as VFSS or FEES. While those procedures are essential for diagnosing a patient’s internal swallowing physiology, our tool is engineered to ensure that the external environment—the mechanical properties of the meal—remains strictly compliant with those clinical findings. By serving as an environmental gatekeeper rather than a diagnostic instrument, the platform closes the safety loop between the physician’s order and the patient’s plate without overstepping critical clinical boundaries.

#### 5.3.1 Methodological positioning: feasibility validation vs. interventional cohort trials

To safely transition this technology to active edge environments, a clear line must be drawn between an architectural feasibility baseline and an interventional human-subject clinical trial. Because exposing a vulnerable, dysphagic patient cohort to automated visual sorting without preliminary safety data carries severe, life-threatening aspiration risks, this study deliberately limits its scope to technical validation and clinical-expert simulation. Rather than gathering direct patient feedback via control and intervention dynamics, our framework utilizes a target-oriented clinical-expert proxy validation paradigm. By benchmarking system precision against physical IDDSI audit protocols executed by medical specialists, we establish a validated, risk-free safety envelope. This technical baseline is a strict ethical prerequisite, serving as the verified launchpad for the multi-center human-subject cohort trials outlined in our future research trajectory.

### 5.4 Addressing the modality–construct gap

The modality–construct gap is a significant technical barrier in the field of visual dietetics and automated clinical screening. IDDSI levels are fundamentally defined by physical behavior under shear and gravitational forces (mechanical constructs), which are inherently difficult to resolve fully from static RGB images (optical constructs).

Critical safety properties—such as internal elasticity, bolus cohesiveness, or the hidden adhesiveness of a puree—may appear visually identical to safe textures in a 2D plane while behaving dangerously during the oral and pharyngeal phases of swallowing (4). This limitation mirrors the challenges documented in current literature regarding single-view food portion estimation, where mapping 2D pixels to 3D physical volumes and mass consistently encounters accuracy ceilings due to the lack of depth data (30, 31). While traditional IoT solutions have addressed nutrition intake monitoring through physical hardware, such as multiscale weighing systems and centralized data aggregation (36), our framework seeks to provide a non-invasive, vision-based alternative.

To mitigate this issue, our intelligent soft sensor framing utilizes CoT prompting to force the model to perform a simulated audit of flow rates, gravitational drip behaviors, and surface patterns. By requiring the model to “reason through” the physical interaction between the food and the utensil, we shift the model from simple pattern matching to a physics-informed heuristic. However, because of the optical–mechanical disconnect, the platform functions as a high-fidelity screening tool rather than a final diagnostic authority. Users must still be encouraged to perform physical verification (e.g., the fork pressure or spoon tilt test) for any borderline cases to ensure absolute patient safety.

### 5.5 Geographical scope and database modularity

A central challenge in evaluating such a system is the current global absence of established public benchmarks for visual IDDSI classification. Our introduction of the CEIV benchmark—where we demonstrated a 9.2% total FNR and a 7.14% FNR for liquid aspiration—is a foundational step toward the standardisation of AI-driven texture screening.

The system is currently optimized for Hong Kong’s specific culinary landscape through integration with the CFS database. However, the platform’s modular retrieval layer can support hot-swapping of regional repositories. This flexibility enables the platform to adapt rapidly to global food environments by mapping the MLLM’s semantic output to alternative taxonomies, such as the USDA FoodData Central (18). By decoupling the intelligent soft sensor logic from regional nutritional metadata, we ensure that the system maintains high cross-cultural transferability and clinical utility without needing the core reasoning pipeline to be redesigned comprehensively.

**Figure 8:**
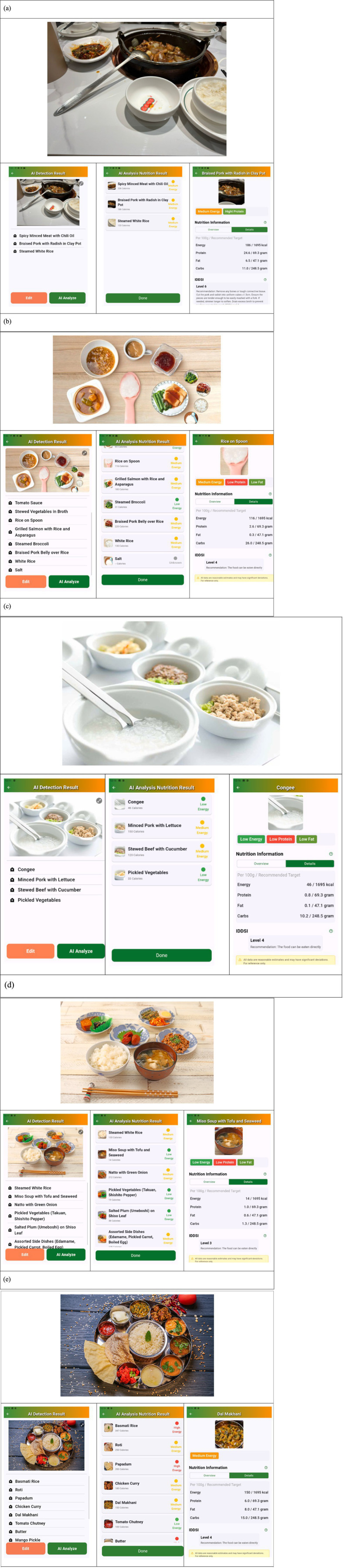
Results across diverse dietary scenarios as visualized by our developed app. **(a)** Normal meal consisting of Chinese food/Hong Kong-style food. **(b, c)** Typical meal for dysphagia. **(d, e)** Out-of-distribution food (Japanese food, Indian food).

Moreover, we tested some out-of-distribution food by using our framework, and the model’s performance across diverse scenarios, including traditional Cantonese dishes and international cuisines, is visualized in Figure 8. The result shows that our proposed framework could identify food of different cultures, demonstrating its strong generalizability.

### 5.6 Future research directions

To evolve the platform from a feasibility study to a flexible IoT-based tool, future research will focus on the following:

- Temporal video analysis: A significant leap in closing the modality–construct gap involves transitioning from static RGB images to short video clips. By analyzing the temporal dynamics of food—specifically the way a liquid flow or a solid rebound under utensil pressure—future iterations can capture the rheological and mechanical properties that are currently inferred via static visual proxies. This approach will shift the system from visual semantic reasoning to dynamic physical analysis.
- Public benchmark expansion: To address the global lack of standardized AI datasets for dysphagia, we aim to expand the CEIV Benchmark into a multicenter, open-access, and expert-annotated repository. This expansion will facilitate global MLLM benchmarking, allowing researchers to evaluate safety-critical models against a diverse range of ethnic cuisines and specialized therapeutic textures.
- Edge-agnostic optimization and privacy: To enhance the system’s operational autonomy, future work will focus on fine-tuning smaller, quantized MLLMs to run natively on mobile hardware. This shift to on-device inference will ensure sub-second latency and absolute data privacy, making the tool suitable for high-security clinical environments and regions with limited network infrastructure.
- Prospective randomized controlled clinical trials: To definitively bridge the technical validation baseline and real-world clinical utility, the ultimate future milestone focuses on executing a formal, human-subject longitudinal cohort study. We plan to launch a controlled clinical trial involving an estimated cohort of 100–120 patients diagnosed with dysphagia secondary to acute stroke or neurodegenerative conditions. The study design will divide participants into an intervention group (where home caregivers utilize the mobile platform for pre-meal texture safety checks) and a control group (relying strictly on traditional visual memory and manual preparation). The evaluation will track comparative outcomes across a 6-month window and measure key metrics to compare the efficiency of our proposed platform and traditional methods.

## 6 Conclusion

This study presents an innovative dietary IoT healthcare platform that addresses the critical safety requirements of patients living with dysphagia. By synergizing MLLMs with a cloud–edge IoT architecture, we successfully used the standard smartphone camera as an intelligent soft sensor. The platform automates a traditionally expert-dependent clinical workflow: localizing meal components, assigning canonical nomenclature, retrieving validated nutrition data from the CFS Hong Kong database, and estimating conservative IDDSI levels with patient-specific texture modification suggestions.

Our framework effectively addresses the persistent nutrition–texture disconnect in prior digital health solutions, which typically prioritize caloric tracking at the expense of mechanical swallowing safety. Through a cascaded three-stage pipeline, the system demonstrates robust operational viability as a feasibility study. Key performance highlights include the following:

- Safety-first performance: Quantitative evaluation against the CEIV Benchmark reveals that the proposed Qwen-based pipeline achieves a superior safety profile compared with leading frontier models.
- Hazard mitigation: The system maintained the lowest total FNR of 9.20%.
- Liquid aspiration detection: Most significantly, while localized open-source models failed entirely to identify liquid hazards (100% FNR), our system remained highly sensitive to aspiration risks with a 7.14% liquid FNR, thus being a reliable first-line screening tool.

**Figure 9:**
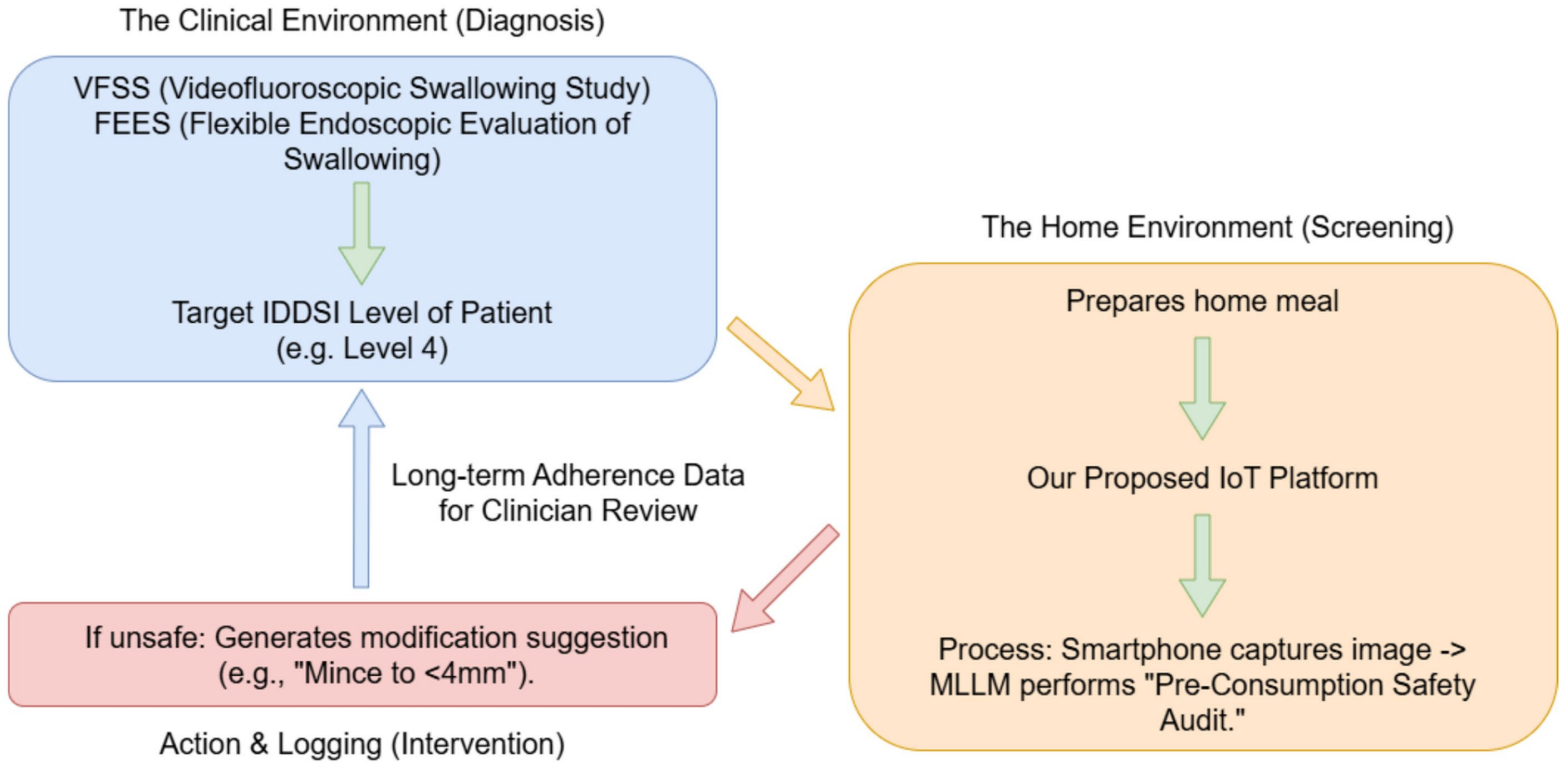
Clinical-to-home workflow.

While inherent limitations remain regarding the modality–construct gap between optical appearance and mechanical rheology, this research provides a scalable foundation for next-generation software sensors in clinical nutrition. By bringing medical-grade IDDSI auditing out of the hospital and into the domestic dining environment, this platform empowers caregivers and patients to manage swallowing safety with significantly higher confidence. The clinical-to-domestic workflow, delineating the boundary between professional diagnosis and home-based screening, is visualized in Figure 9. Ultimately, this technology serves as a critical safety bridge, potentially reducing the incidence of aspiration-related complications and improving the quality of life for the global aging and post-stroke population.

## Data Availability

The original contributions presented in the study are included in the article/supplementary material, further inquiries can be directed to the corresponding author.
